# Supplementary material for: Observation of Boyer-Wolf Gaussian modes
Source: Nat Commun. 2024 Jun 21;15:5301. doi: 10.1038/s41467-024-49456-x (PMC11192905; doi:10.1038/s41467-024-49456-x)
Supplement: Supplementary file 1 — Supplementary Information [file 41467_2024_49456_MOESM1_ESM.pdf]

Supplementary Information

Observation of Boyer-Wolf Gaussian Modes

Konrad Tschernig†, David Guacaneme†, Oussama Mhibik, Ivan Divliansky, Miguel A. Bandres

CREOL, The College of Optics and Photonics, the University of Central Florida, Orlando, FL, USA.

†These authors contributed equally to this work.

Supplementary Note 1: Hamiltonian Description of Optical Resonators

The evolution of rays of light in an optical resonator is most generally described by an ABCD full round-trip matrix [1]

$$M = \begin{pmatrix} A & B \\ C & D \end{pmatrix}, \tag{1}$$

which maps a ray-vector $\vec{\mu} = (x, s_x)^T$ at the transverse position x with slope s_x to the new ray

$$\begin{pmatrix} x' \\ s'_x \end{pmatrix} = \begin{pmatrix} A & B \\ C & D \end{pmatrix} \begin{pmatrix} x \\ s_x \end{pmatrix}. \tag{2}$$

In general, the matrix M is composed of a product of ABCD matrices $M = \prod_j M_j$, where the M_j correspond to the different optical elements of the cavity, such as free space propagation, lenses and mirrors. As shown in Ref. [2] it is possible to map these discrete ray dynamics to a continuous Hamiltonian picture. For the sake of simplicity, and what is most relevant for our discussion, we will consider a Fabry-Pérot resonator. In our case, the cavity consists of two mirrors - one flat and one spherical with radius R - that are separated by the distance L, as seen in Supplementary Fig. (1). The ABCD-matrix of the round-trip is then

$$M = \begin{pmatrix} 1 & L \\ 0 & 1 \end{pmatrix} \begin{pmatrix} 1 & 0 \\ -2/R & 1 \end{pmatrix} \begin{pmatrix} 1 & L \\ 0 & 1 \end{pmatrix} = \begin{pmatrix} 2g-1 & 2gR(1-g) \\ -2/R & 2g-1 \end{pmatrix} \tag{3}$$

where $g = 1 - L/R$. Crucially, this matrix resembles a rotation matrix

$$R = \begin{pmatrix} \cos\alpha & \sin\alpha \\ -\sin\alpha & \cos\alpha \end{pmatrix}. \tag{4}$$

In order to match R and M we need to introduce a coordinate scaling

$$\beta = \begin{pmatrix} 1 & 0 \\ 0 & \omega \end{pmatrix}, \tag{5}$$

which yields

$$\widetilde{R} = \beta R \beta^{-1} = \begin{pmatrix} \cos(\alpha) & \sin(\alpha)/\omega \\ -\omega \sin(\alpha) & \cos(\alpha) \end{pmatrix}. \tag{6}$$

By comparing M and $\widetilde{R}$ we find immediately

$$\omega = \frac{1}{R\sqrt{g(1-g)}} = \left[L\left(R-L\right)\right]^{-1/2}. \tag{7}$$

Since the generator of rotation is the Pauli σ_y matrix we can thus write M as

$$M = \widetilde{R} = \exp\left(-i\alpha\beta\sigma_y\beta^{-1}\right) = \exp\left[z\begin{pmatrix} 0 & 2 \\ -2\omega^2 & 0 \end{pmatrix}\right], \tag{8}$$

where we redefined $\alpha \to z\omega/2$. This result suggests that the continuous equation of motion for the ray-vector $\vec{\mu}$ along the synthetic time dimension z is

$$\partial_z \vec{\mu}(z) = Q\vec{\mu}(z) \text{ with } Q = \begin{pmatrix} 0 & 2 \\ -2\omega^2 & 0 \end{pmatrix}. \tag{9}$$

Explicitly, we arrive at the set of coupled differential equations

$$\dot{x} = 2s_x \qquad \dot{s}_x = -2\omega^2 x. \tag{10}$$

Using Hamilton's equations

$$\dot{x} = \frac{\partial H}{\partial s_x} \qquad \dot{s}_x = -\frac{\partial H}{\partial x}, \tag{11}$$

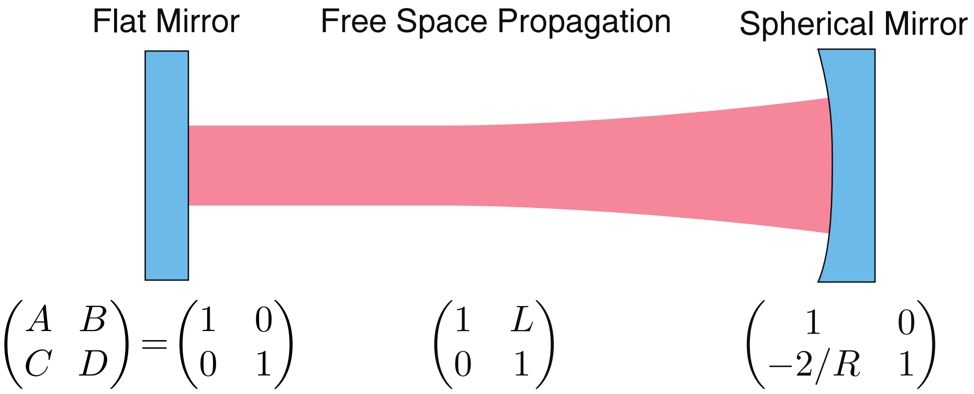

Supplementary Fig. 1 The Fabry-Pérot resonator. The cavity consists of two mirrors, one flat and one spherical with radius R, separated by length L. The flat mirror is semi-reflective and acts as the laser output and as the reference plane of the cavity roundtrip. Below we show the ABCD matrix of the corresponding optical elements.

we arrive at the Hamiltonian representation of the Fabry-Pérot resonator

$$H = s_x^2 + \omega^2 x^2. \tag{12}$$

Therefore, the Fabry-Pérot resonator with spherical mirror radius R and length L is mapped to a harmonic oscillator trap potential with strength $\omega = [L(R-L)]^{-1/2}$ and x is the position of the 'trapped particle'.

The slope s_x of the ray is proportional to the transverse momentum $p_x = ks_x$ of the particle, where $k = 2\pi/\lambda$ is the wave number. Thus, by substituting $s_x \to -i\partial_x/k$ we find the eigenvalue equation for the eigenmodes $\psi(x)$ of the resonator

$$-\frac{\partial^2\psi}{\partial x^2} + k^2\omega^2 x^2\psi = E\psi. \tag{13}$$

This leads to the well-known result that the Fabry-Pérot resonator supports Hermite-Gauss eigenmodes

$$\psi_n(x) = \left[2^{n-1/2} n! \sqrt{\pi} w_0\right]^{-1/2} e^{-x^2/w_0^2} H_n\left(\sqrt{2}\frac{x}{w_0}\right) \tag{14}$$

$$E_n = \frac{2}{w_0^2}(2n+1). \tag{15}$$

Here we defined the $1/e^2$ mode waist at the plane of the output flat mirror

$$w_0^2 = \frac{2}{\omega k} = \frac{2}{k}\sqrt{L(R-L)}, \tag{16}$$

which is also the plane we selected to start the full cavity-roundtrip.

Supplementary Note 2: Design of the Boyer-Wolf-Gaussian Cavity

In the previous section we have seen that the modes of a Fabry-Pérot resonator are described by a harmonic oscillator potential with strength $\omega = [L(R-L)]^{-1/2}$. Our goal is now to design a resonator to implement a 2D harmonic oscillator potential

$$H = -\partial_x^2 - \partial_y^2 + k^2\omega_x^2 x^2 + k^2\omega_y^2 y^2. \tag{17}$$

with the specific ratio $\omega_x = 2\omega_y$. The first intuitive idea would be to replace the spherical mirror in Supplementary Fig. (1) with an astigmatic mirror with the radii R_x and R_y in the x- and y-direction, respectively. Then we need to choose R_y, such that $\omega_x = [L(R_x - L)]^{-1/2} = 2\omega_y = 2[L(R_y - L)]^{-1/2}$, which is achieved by $R_y = 4R_x - 3L$. However, such a design has two major drawbacks - firstly, astigmatic mirrors are quite challenging to manufacture and secondly, the resonator would only exhibit the desired 2:1 ratio for one specific distance of the mirrors, which further complicates the assembly of such a resonator.

Here we propose an elegant design that solves both of these issues. Firstly, we begin with the design shown in Supplementary Fig. (1) and instead of a spherical mirror we use a cylindrical mirror in y-direction. As we have seen, this leads to a harmonic oscillator potential in y-direction with

$$\omega_y = \left[L\left(R_y - L\right)\right]^{-1/2}. \tag{18}$$

For now the potential in x-direction has strength $\omega_x = 0$, since the cylindrical mirror has the radius $R_x = \infty$ in x-direction. However, this crucial step enables us to decouple the x and y degrees of freedom and to tune ω_x and ω_y separately. To do so, we first emphasize the formal equivalence between lenses and mirrors within the ABCD-framework, such that a mirror of radius R is equivalent to a lens with focal length $f = R/2$. The idea is now to place a cylindrical lens with focal length f_x - corresponding to a cylindrical mirror with $R_x = 2f_x$ - in the center of the cavity at distance $L_x = L/2$, as shown in Supplementary Fig. (2). Thus, in x-direction the Fabry-Pérot resonator exhibits the strength of the corresponding harmonic oscillator potential

$$\omega_x = \left[L/2\left(2f_x - L/2\right)\right]^{-1/2} = 2\left[L(4f_x - L)\right]^{-1/2} = 2\omega_y. \tag{19}$$

Note, that one roundtrip of the whole resonator corresponds to two Fabry-Pérot-roundtrips in the x-direction of the resonator. Therefore we can achieve the desired 2:1 ratio by choosing $4f_x = R_y = 2R_x$ and obtain the 2D Hamiltonian

$$H = -\partial_x^2 - \partial_y^2 + k^2\omega^2(4x^2 + y^2), \tag{20}$$

with

$$\omega = \left[L\left(R_y - L\right)\right]^{-1/2}. \tag{21}$$

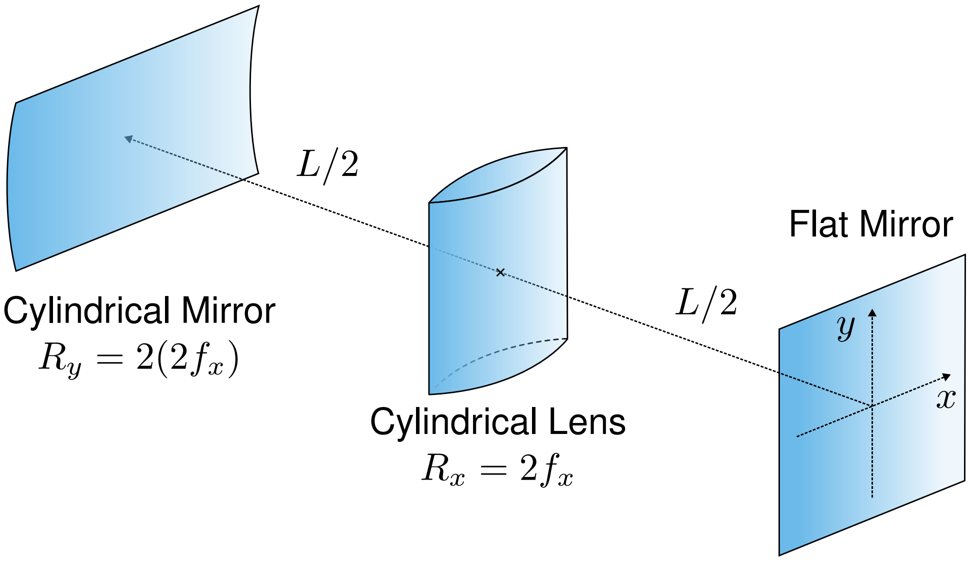

Supplementary Fig. 2 Sketch of the resonator to generate Boyer-Wolf Gaussian beams. Starting from the Fabry-Pérot resonator in Supplementary Fig. (1), we have added a cylindrical lens of focal length f_x in the center of the cavity and replaced the spherical mirror with a cylindrical one with radius $R_y = 4f_x$. This ensures that the effective oscillator strengths in the Hamiltonian-picture of the cavity obey the ratio $\omega_x = 2\omega_y$. One roundtrip in the y-direction now corresponds to two roundtrips in the x-direction.

Supplementary Note 3: Boyer-Wolf Gaussian Modes

In this section we find the Boyer-Wolf-Gaussian eigenmodes of the 2:1 resonator

$$\hat{H}\psi = (-\partial_x^2 - \partial_y^2)\psi + k^2\omega^2(4x^2 + y^2) = E\psi. \tag{22}$$

We proceed to solve this eigenvalue problem as shown in ref. [3]. Firstly, there are two unique operators that commute with $\hat{H}$ [3]

$$\hat{S}_C = -\partial_x^2 + 4k^2\omega^2 x^2 \tag{23}$$

$$\hat{S}_P = x\partial_x^2 - y\partial_x\partial_y - \frac{1}{2}\partial_x + k^2\omega^2 xy^2 \tag{24}$$

which are associated with Cartesian coordinates (C) and Parabolic coordinates (P), respectively. Thus, there are two equivalent representations of the eigenmodes, which stem from diagonalizing either the pair $(\hat{H}, \hat{S}_C)$ or the pair $(\hat{H}, \hat{S}_P)$. From the first pair it is straight-forward to see that $\phi = \phi_1(x)\phi_2(y)$ factorizes, and we obtain the 2D harmonic oscillator modes in terms of the Hermite-Gauss polynomials

$$HG_{n_1,n_2}(x,y) = \left[2^{n_1+n_2-3/2}\pi n_1! n_2! w_0^2\right]^{-1/2} \exp\left(-\frac{2x^2+y^2}{w_0^2}\right) H_{n_1}\left(2\frac{x}{w_0}\right) H_{n_2}\left(\sqrt{2}\frac{y}{w_0}\right) \tag{25}$$

$$E = \frac{2}{w_0^2}(4n_1 + 2n_2 + 3), \qquad \mu = \frac{8}{w_0^2}(n_1 + 1/2), \tag{26}$$

where $\hat{S}_C HG_{n_1,n_2} = \mu HG_{n_1,n_2}$ and the mode width is given by

$$w_0^2 = \frac{2}{\omega k} = \frac{\lambda}{\pi}\sqrt{L(R_y - L)}. \tag{27}$$

We emphasize that w_0 is the $1/e^2$ mode waist at the plane of the flat output mirror, which defines the start of the full cavity roundtrip.

In a similar but slightly more elaborate way the wavefunction also factorizes in parabolic coordinates (u, v) [3, 4], where

$$x = \frac{1}{2}(u^2 - v^2) \qquad y = uv, \tag{28}$$

and their domains are given by $u \in (-\infty, \infty)$ and $v \in [0, \infty)$. In parabolic coordinates, (u, v), the Hamiltonian now takes the form

$$\hat{H} = -\frac{1}{u^2 + v^2}\left(\partial_u^2 + \partial_v^2\right) + k^2\omega^2\left(u^4 - u^2v^2 + v^4\right). \tag{29}$$

Thus we can write the eigenvalue equation $\hat{H}\phi = E\phi$ in the following way

$$\left(-\partial_u^2 - \partial_v^2 + k^2\omega^2 u^6 + k^2\omega^2 v^6 - Eu^2 - Ev^2\right)\phi = \widetilde{H}\phi = 0. \tag{30}$$

By applying the separation of variables ansatz $\phi(u, v) = \Phi_1(u)\Phi_2(v)$ we find

$$\left(-\partial_u^2 + k^2\omega^2 u^6 - Eu^2\right)\Phi_1(u) = \mu\Phi_1(u) \tag{31}$$

$$\left(-\partial_v^2 + k^2\omega^2 v^6 - Ev^2\right)\Phi_2(v) = -\mu\Phi_2(v), \tag{32}$$

where μ is the separation constant. Using the transformation $v \to iv$ we see that Eq. (31) transforms into Eq. (32) exactly and thus Φ_1 and Φ_2 are in fact identical functions. Thus, our ansatz can be modified to $\phi(u,v) = \Phi(u)\Phi(iv)$ where $\Phi(u)$ is the solution of the eigenvalue problem

$$\left(-\partial_u^2 + k^2\omega^2 u^6 - Eu^2\right)\Phi(u) = \mu\Phi(u). \tag{33}$$

This is a special case of the eigenvalue equation of the sextic harmonic oscillator [5–9] and we study the solutions in the section 1. Note, the factorization in parabolic coordinates is only possible due to the 2:1 ratio of the oscillator strengths, while the factorization in cartesian coordinates is possible for any ratio.

Following Ref. [3] we find the Boyer-Wolf Gaussian eigenmodes of the anisotropic 2:1 oscillator, Eq. (22)

$$BWG_{nl}(u,v) = c_{nl}\left(\sqrt{2}\frac{uv}{w_0}\right)^n \exp\left(-\frac{u^4+v^4}{2w_0^2}\right) P_{nl}\left(\left[4\frac{u^2}{w_0}\right]^{-1}\right) P_{nl}\left(-\left[4\frac{v^2}{w_0}\right]^{-1}\right), \tag{34}$$

where $w_0^2 = \lambda/\pi\sqrt{L(R_y - L)}$. The solutions are characterized by the mode numbers $n = 0, 1, 2\ldots$ and $-\frac{1}{2}\lfloor n/2\rfloor \le l \le \frac{1}{2}\lfloor n/2\rfloor$ and the parabolic polynomials $P_{nl}(x)$ which we discuss in section 1. Note, $\lfloor x\rfloor$ denotes the integer part of x.

We show several examples of the Boyer-Wolf modes in Supplementary Figs. (3-6). In Supplementary Figs. (3,4) we show the amplitude distributions of the Boyer-Wolf modes up to $n \le 9$. Note, the mode order n corresponds to the eigenvalue

$$E_n = 2(2n+3)/w_0^2, \tag{35}$$

which has degeneracy of $\lfloor n/2\rfloor + 1$. The mode index l enumerates the degenerate eigenmodes of order n and is arranged in such a way that modes of opposite sign $l \leftrightarrow -l$ are mirrored versions of each other

$$BWG_{n,l}(x,y) = BWG_{n,-l}(-x,y). \tag{36}$$

We remark that the total number of nodal lines, where the mode field vanishes, is given by

$$m = \left\lfloor \frac{n+1}{2} \right\rfloor. \tag{37}$$

As a consequence, for example, the set of modes BWG_{5l} and BWG_{6l} feature the same number of nodal lines $m = 3$, which can be seen in Supplementary Figs. (3,4). For odd n, the x-axis is always a nodal line, while all other nodal lines are parabolas, that are open either to the positive or negative x-direction. The number of positive m_+ and negative m_- nodal lines is given by

$$m_+ = l + \left\lfloor\frac{n}{2}\right\rfloor\frac{1}{2} \qquad m_- = \left\lfloor\frac{n}{2}\right\rfloor\frac{1}{2} - l. \tag{38}$$

Further, we show the intensity distributions of Boyer-Wolf modes in Supplementary Figs. (5,6). The normalization constants c_{nl} in Eq. (34) are given by

$$c_{nl} = \left(p^{nl}_{\lfloor n/2 \rfloor}\right)^{-2} \pi^{-1/2} 2^{n+(-1)^n/4} \sum_{m=0}^{\lfloor n/4 \rfloor} p^{nl}_{2m} (-1)^m \frac{(2m)!(n-4m)!}{m!(\lfloor n/2 \rfloor - 2m)!}, \tag{39}$$

and the coefficients p^{nl}_m will be discussed in section 1. We note a useful relation [3] which enables the decomposition of the Boyer-Wolf Gaussian modes into superpositions of Hermite-Gauss modes

$$BWG_{nl}(x,y) = \sum_{m=0}^{\lfloor n/2 \rfloor} \sqrt{2^{-m} m!(n-2m)!} p^{nl}_m HG_{m,n-2m}(x,y). \tag{40}$$

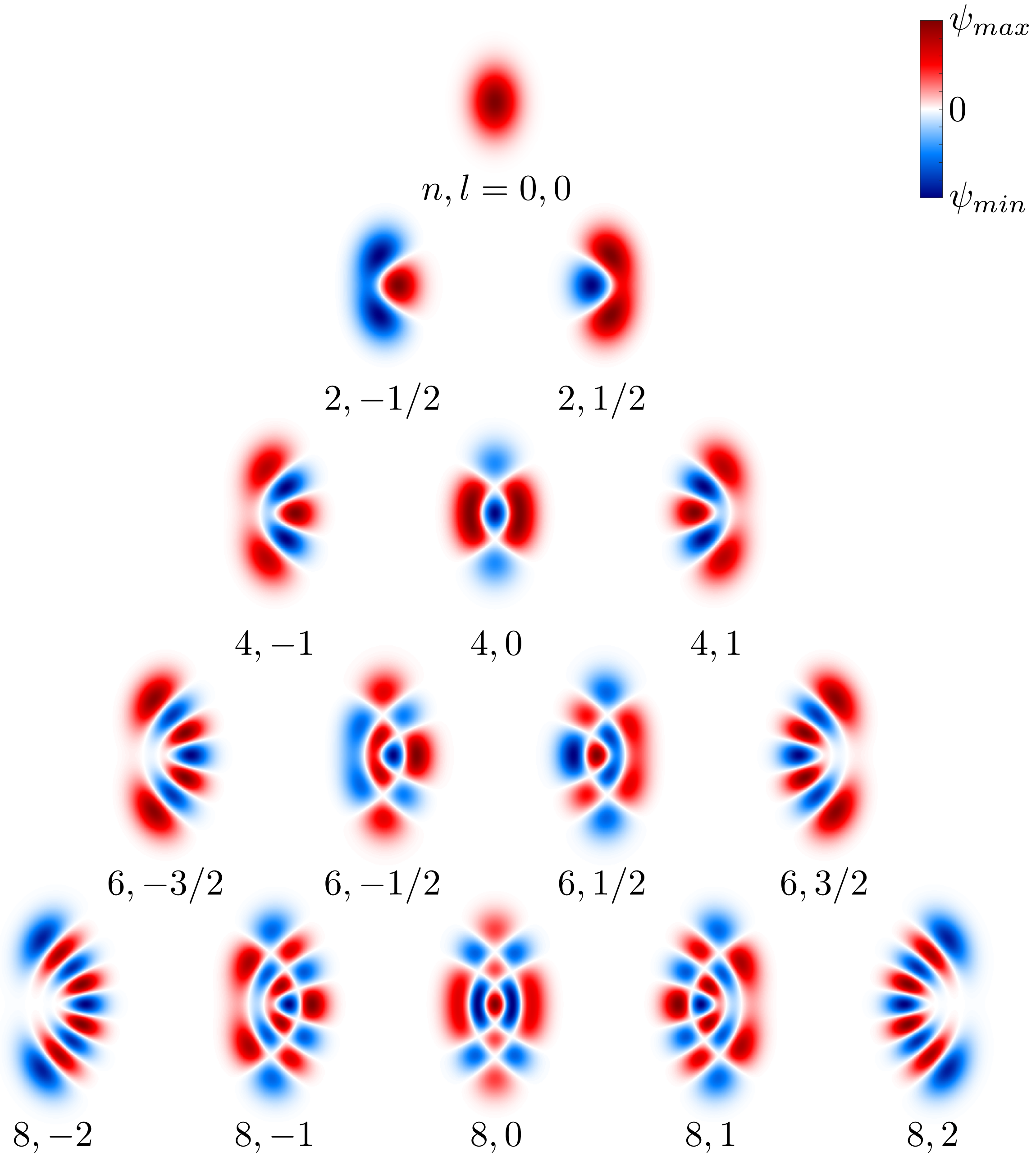

Supplementary Fig. 3 Even Boyer-Wolf-Gaussian modes. The field distribution of the first 15 Boyer-Wolf-Gaussian modes $BWG_{nl}(x, y)$ with even n, where blue (red) indicate negative (positive) real amplitudes. Observe that the transformation $l \to -l$ implies mirroring the mode along the y-axis.

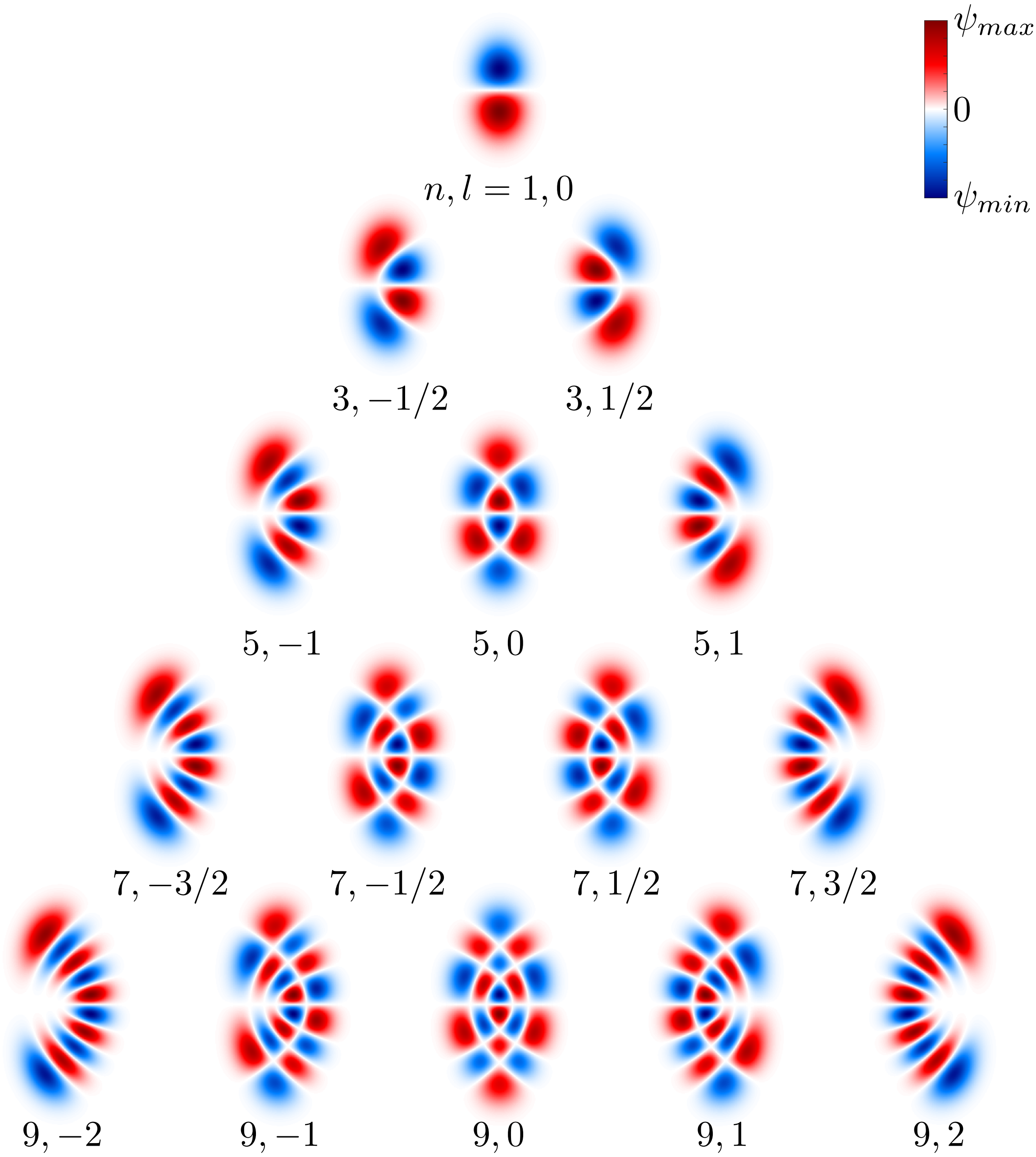

Supplementary Fig. 4 Odd Boyer-Wolf-Gaussian modes. The field distribution of the first 15 Boyer-Wolf-Gaussian modes $BWG_{nl}(x, y)$ with odd n, where blue (red) indicate negative (positive) real amplitudes. Observe that the transformation $l \to -l$ implies mirroring the mode along the y-axis.

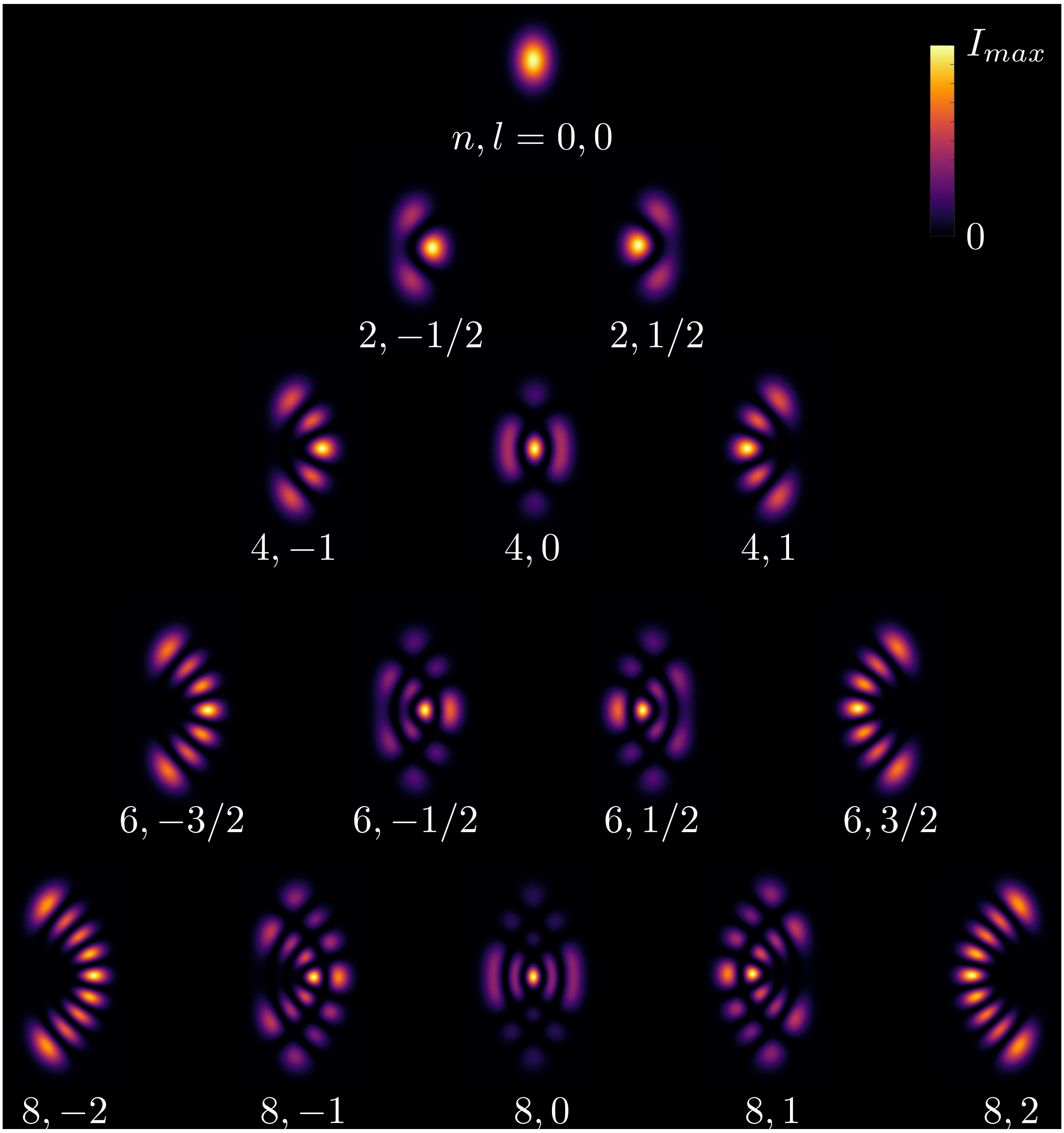

Supplementary Fig. 5 Even Boyer-Wolf-Gaussian modes. The intensity distribution of the first 15 Boyer-Wolf-Gaussian modes $|BWG_{nl}(x, y)|^2$ with even n. Observe that the transformation $l \to -l$ implies mirroring the mode along the y-axis.

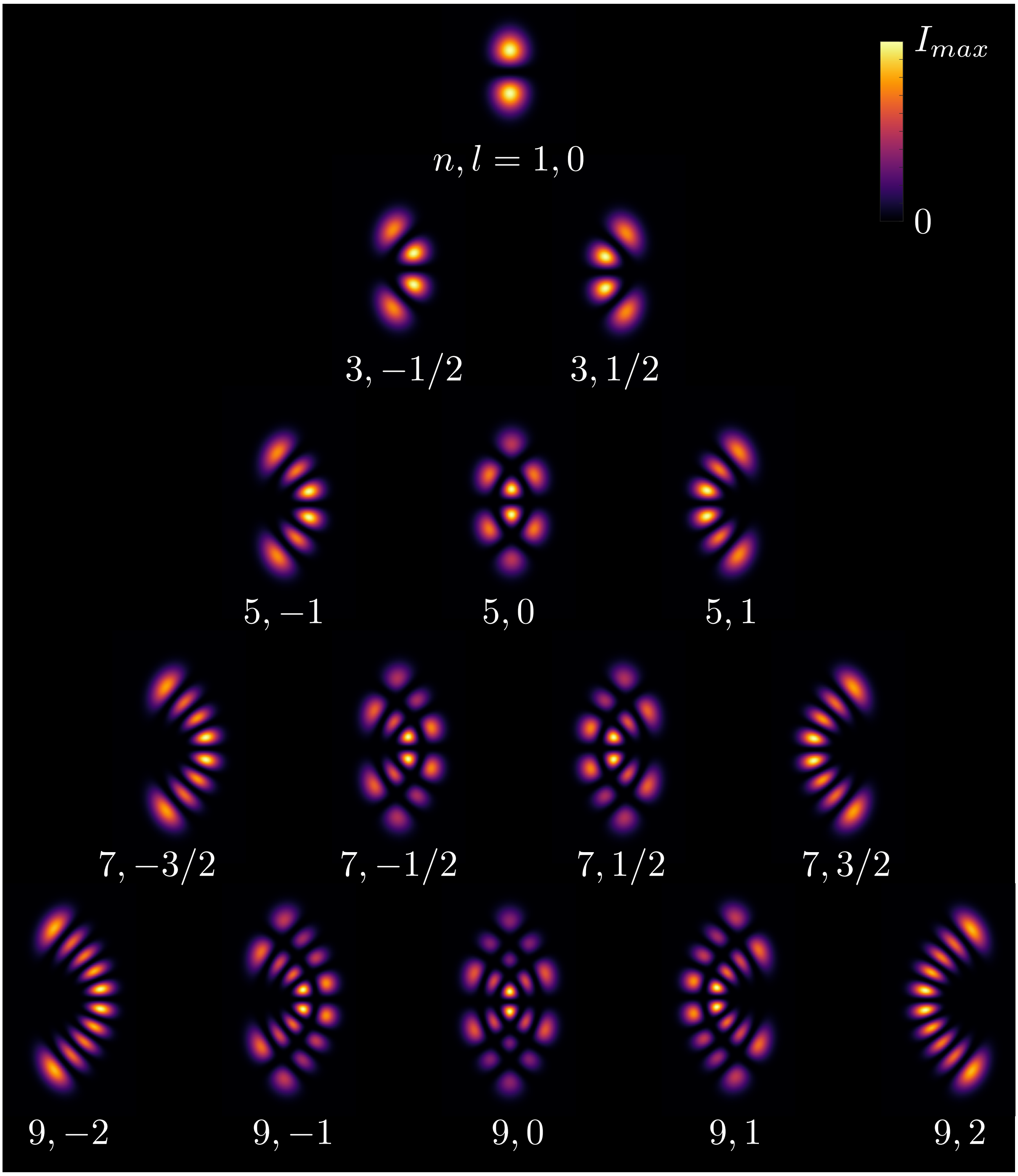

Supplementary Fig. 6 Odd Boyer-Wolf-Gaussian modes. The intensity distribution of the first 15 Boyer-Wolf-Gaussian modes $|BWG_{nl}(x, y)|^2$ with odd n. Observe that the transformation $l \to -l$ implies mirroring the mode along the y-axis.

Supplementary Note 4: Propagation of the BWG Modes in the Optical Resonator

In Supplementary Fig. (7) we show simulations of the propagation of the Boyer-Wolf-Gaussian beams in the 2:1 laser cavity. Specifically we show the unfolded propagation [1] by replacing the cylindrical mirror R_y with an equivalent cylindrical lens $f_y = R_y/2$. Clearly, the Boyer-Wolf-Gaussian modes are proper eigenmodes of the 2:1 anisotropic Fabry-Pérot resonator.

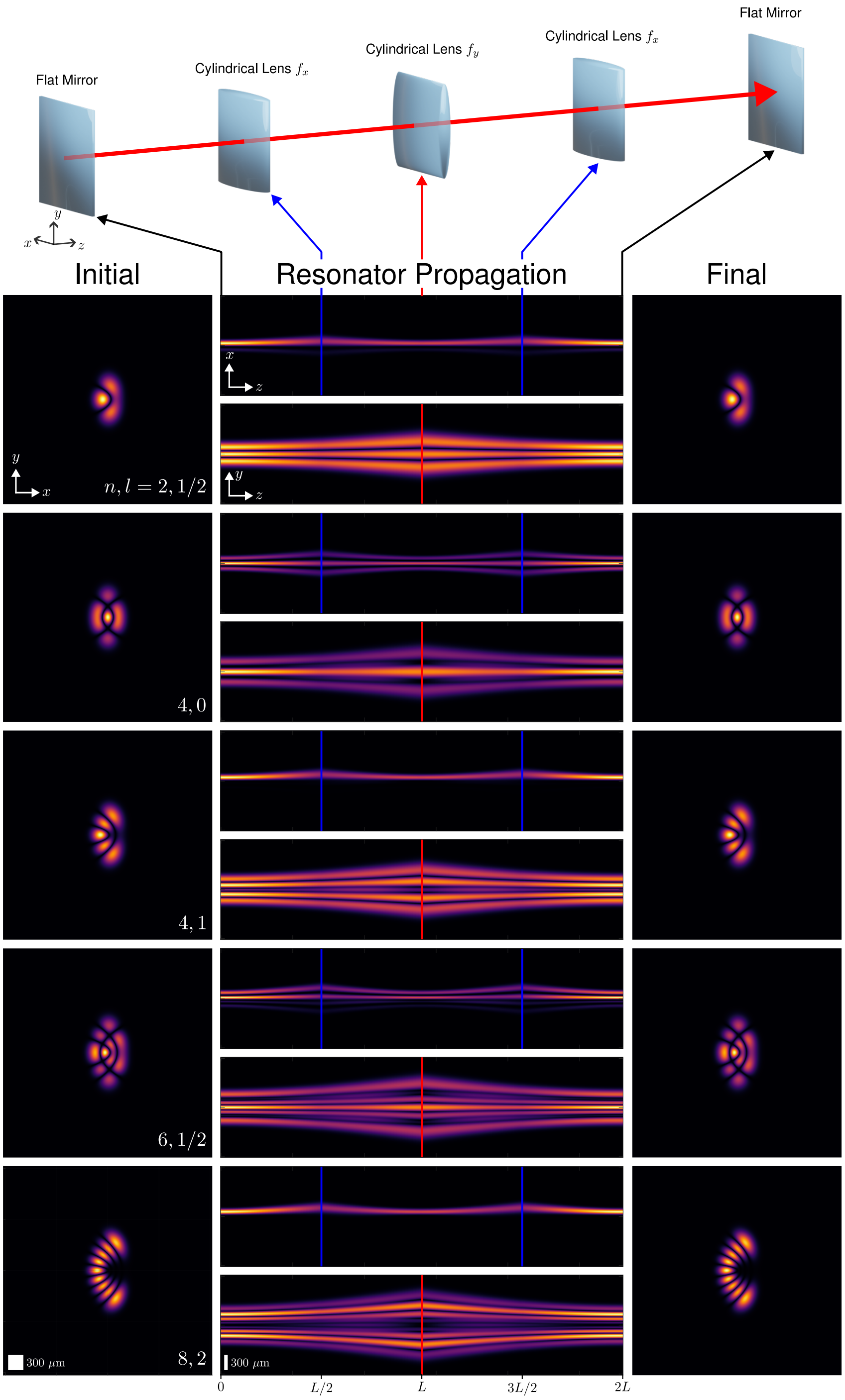

Supplementary Fig. 7 Simulation of the propagation of Boyer-Wolf Gaussian modes in the resonator. The left-most panels show the initial field amplitudes, the center panels the $x - z$ and $y - z$-slice of the propagation and the right-most panel shows the final field amplitudes. In the simulations we choose $\lambda = 1064$ nm, $L = 280$ mm, $f_x = 100$ mm, $R_y = 400$ mm and the initial mode-width $w = 176.2$ μm. The blue (red) vertical lines indicate the positions of the cylindrical lenses in x-(y-)direction. On the top we show a sketch of the unfolded resonator.

Supplementary Note 4.1: Stability

In order to analyze the stability of our resonator setup, it is important to realize that it consists of two Fabry-Pérot-like resonators in x- and y-direction respectively. To be precise, in y-direction the cavity is closed by a flat mirror and a cylindrical mirror with radius $R_y = 4f_x$ at distance L. The stability in

y-direction is therefore governed by the inequality [1]

$$0 < \left(1 - \frac{L}{4f_x}\right)^2 < 1. \tag{41}$$

In x-direction the cavity is closed by two flat mirrors at distance L with a cylindrical lens $R_x = 2f_x$ at $L/2$. This configuration can be folded onto an equivalent cavity that is closed by a flat mirror and a cylindrical mirror $R_x = 2f_x$ at distance $L/2$ and therefore follows the stability criterion

$$0 < \left(1 - \frac{L/2}{2f_x}\right)^2 = \left(1 - \frac{L}{4f_x}\right)^2 < 1. \tag{42}$$

Evidently, the criteria in x- and y- direction are identical and thus the complete cavity is stable if $L < 4f_x = 2R_y$, which we show in Supplementary Fig. (8).

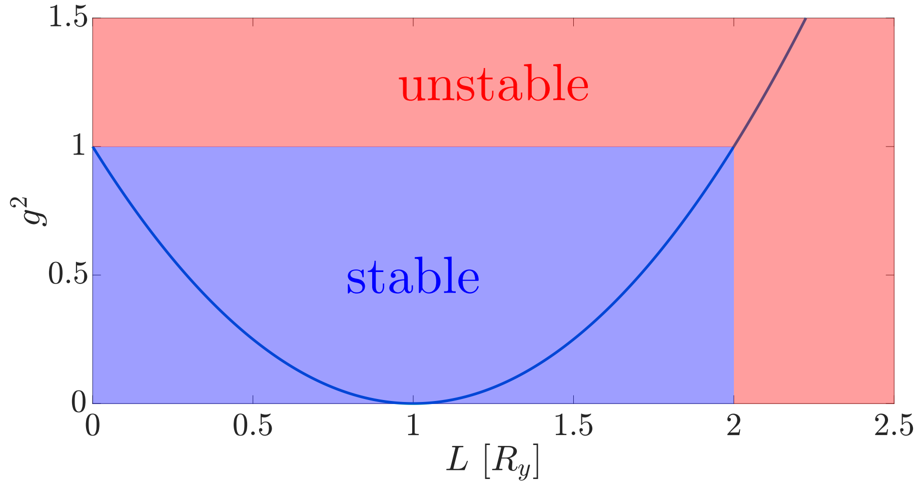

Supplementary Fig. 8 Stability of the Boyer-Wolf cavity. We plot the stability parameter $g^2 = \left(1 - \frac{L}{2R_y}\right)^2$ as a function of the length of the resonator L in units of the radius of the cylindrical mirror $R_y = 2(2f_x)$.

Supplementary Note 4.2: Beam Waist

The beam waist w_0 at the output mirror is given by

$$w_0^2 = \frac{2}{\omega k} \Leftrightarrow w_0^2 = \frac{\lambda}{\pi}\sqrt{L(R_y - L)}, \tag{43}$$

which we show as a function of L ($k = 2\pi/\lambda$, $\lambda = 1064$ nm) in Supplementary Fig. (9). The maximum beam waist is then $w_0(L = R_y/2) = \sqrt{\lambda R_y/(2\pi)}$.

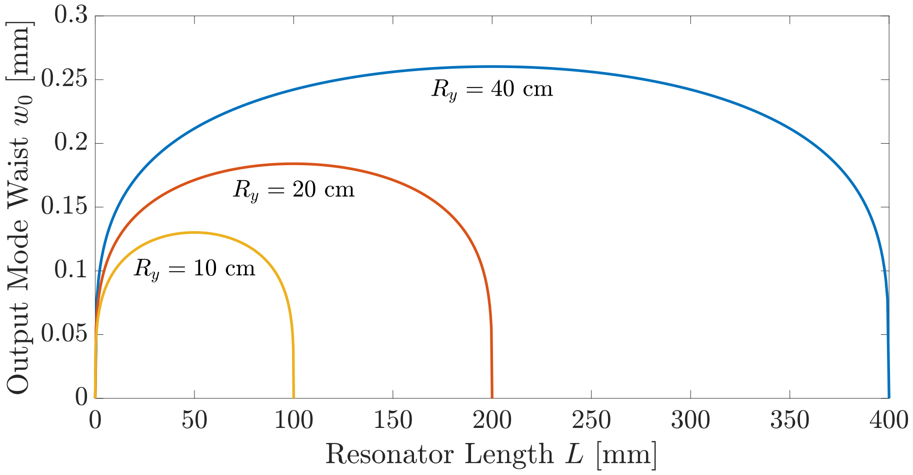

Supplementary Fig. 9 Beam waist of the Boyer-Wolf Gaussian modes. We plot the beam waist width $w_0 = \sqrt{2\sqrt{L(R_y - L)}/k}$ as a function of the length of the resonator L ($k = 2\pi/\lambda$, $\lambda = 1064$ nm).

Supplementary Note 4.3: Fourier Transform of the Boyer-Wolf Gaussian Modes

The Boyer-Wolf-Gaussian modes can be expressed as [3]

$$BWG_{nl}(x,y) = \sum_{m=0}^{\lfloor n/2 \rfloor} \left[2^{-m} m!(n-2m)!\right]^{1/2} p_m^{nl} HG_{m,n-2m}(x,y)\,, \tag{44}$$

where

$$HG_{n_1,n_2}(x,y) = \left[2^{n_1+n_2-3/2}\pi n_1! n_2! w^2\right]^{-1/2} \exp\left(-\frac{2x^2+y^2}{w^2}\right) H_{n_1}\left(2\frac{x}{w}\right) H_{n_2}\left(\sqrt{2}\frac{y}{w}\right), \tag{45}$$

and $w^2 = w_0^2\left(1+\frac{z^2}{z_R^2}\right)$ is the z-dependent beam width (squared), $w_0^2 = \frac{2}{k}\sqrt{L(R_y - L)}$ and $z_R = \pi w_0^2/\lambda$ is the Rayleigh length. The Fourier transform of the BWG modes is then

$$\mathcal{F}[BWG_{nl}](k_x,k_y) = \sum_{m=0}^{\lfloor n/2 \rfloor} \left[2^{-m} m!(n-2m)!\right]^{1/2} p_m^{nl} \mathcal{F}[HG_{m,n-2m}](k_x,k_y)\,. \tag{46}$$

Therefore we need the Fourier Transform of the HG modes, which is a straight-forward task

$$\begin{aligned}\mathcal{F}[HG_{n_1,n_2}](k_x,k_y) = \left[2^{n_1+n_2-3/2}\pi n_1! n_2! w^2\right]^{-1/2} \frac{w^2}{2\sqrt{2}}(-i)^{n_1+n_2} \exp\left(-\frac{w^2k_x^2}{8} - \frac{w^2k_y^2}{4}\right) \\ H_{n_1}\left(\frac{wk_x}{2}\right) H_{n_2}\left(\frac{wk_y}{\sqrt{2}}\right).\end{aligned} \tag{47}$$

In Supplementary Figs. (11,10) we show several examples of the Fourier Transform of the BWG modes in (k_x, k_y)-space.

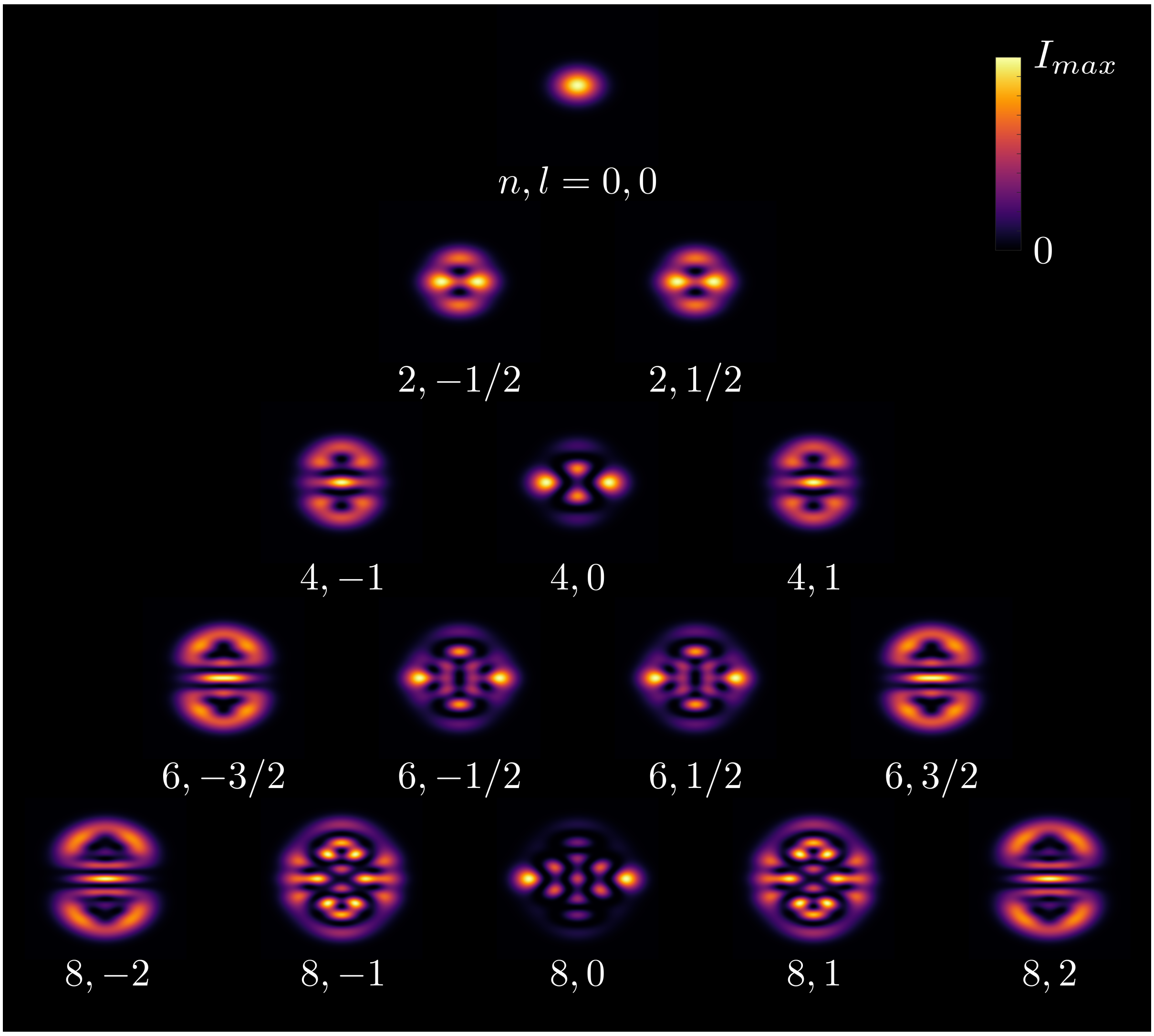

Supplementary Fig. 10 Fourier Transform of the even Boyer-Wolf-Gaussian modes. The intensity distribution of the first 15 Fourier-transformed Boyer-Wolf-Gaussian modes $|\mathcal{FT}\{BWG_{nl}\}(k_x, k_y)|^2$ with even n.

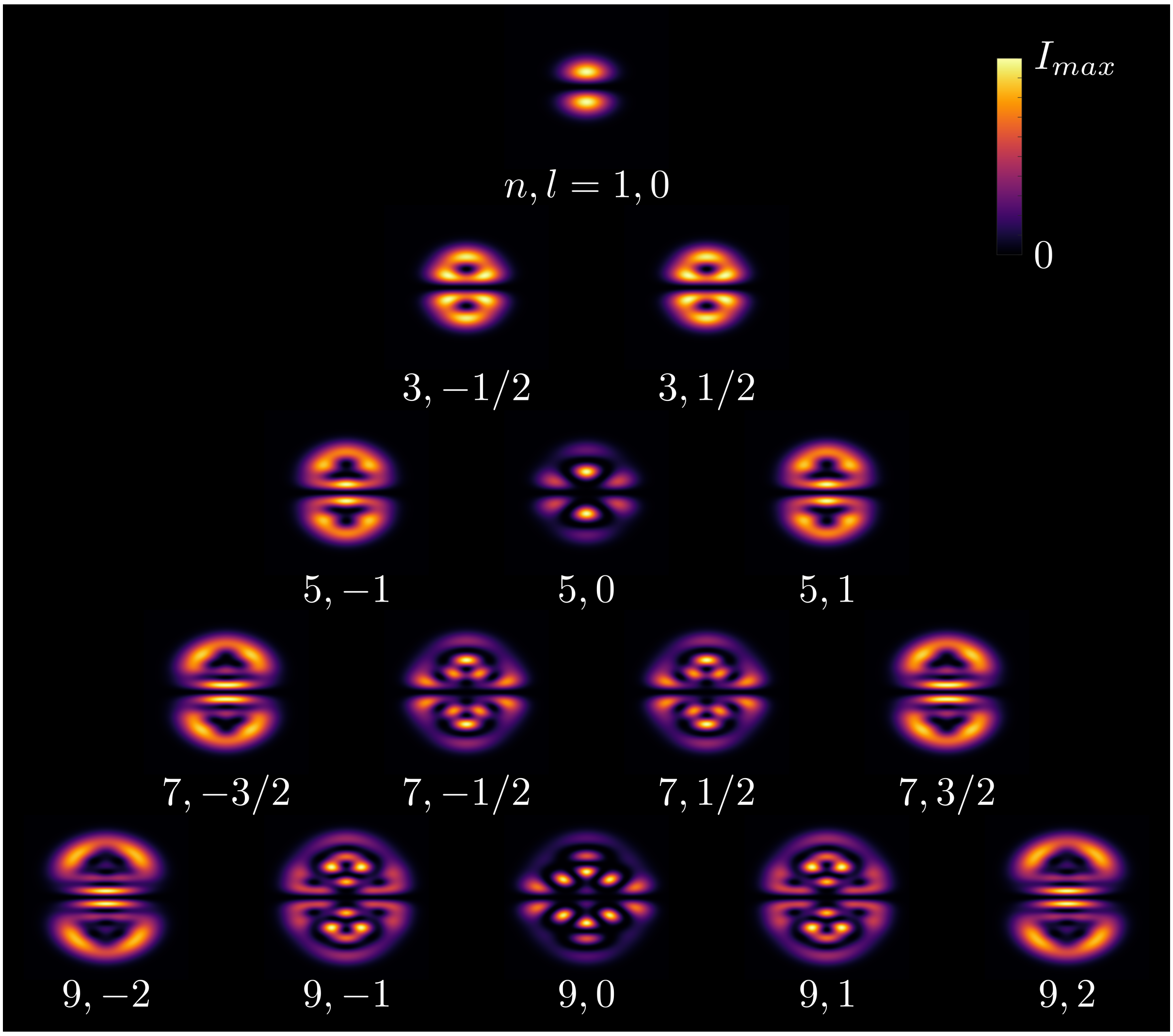

Supplementary Fig. 11 Fourier Transform of the odd Boyer-Wolf-Gaussian modes. The intensity distribution of the first 15 Fourier-transformed Boyer-Wolf-Gaussian modes $|\mathcal{FT}\left\{BWG_{nl}\right\}(k_x, k_y)|^2$ with odd n.

Supplementary Note 4.4: Gouy Phase Shift of the Boyer-Wolf Gaussian Modes

In general, the Gouy phase shift is a result of the spread of the transverse momentum components in a given beam during propagation in direction z [10]

$$\phi_G = -\frac{1}{k}\int^z \langle k_x^2\rangle + \langle k_y^2\rangle \, \mathrm{d}z, \tag{48}$$

where $k^2 = k_x^2 + k_y^2 + k_z^2$, and the average $\langle\cdot\rangle$ is to be taken over the beam field ψ interpreted as a probability distribution

$$\langle\xi\rangle = \frac{\int_{-\infty}^{\infty} \xi|\psi(\xi)|^2\mathrm{d}\xi}{\int_{-\infty}^{\infty} |\psi(\xi)|^2\mathrm{d}\xi}. \tag{49}$$

Using the results from the previous section, we now evaluate

$$\langle k_x^2\rangle = \iint_{-\infty}^{\infty} k_x^2 \left|\mathcal{F}[BWG_{nl}](k_x,k_y)\right|^2 \mathrm{d}k_x\mathrm{d}k_y \tag{50}$$

$$= \sum_{m,m'=0}^{\lfloor n/2\rfloor} \left[2^{-m}m!(n-2m)!\right]^{1/2}\left[2^{-m'}m'!(n-2m')!\right]^{1/2} p_m^{nl}\left(p_{m'}^{nl}\right)^* \tag{51}$$

$$\iint_{-\infty}^{\infty} k_x^2\mathcal{F}[HG_{m,n-2m}](k_x,k_y)\left(\mathcal{F}[HG_{m',n-2m'}](k_x,k_y)\right)^* \mathrm{d}k_x\mathrm{d}k_y, \tag{52}$$

where we already used the fact, that the Boyer-Wolf Gaussian modes are properly normalized, $\int_{-\infty}^{\infty}|\mathcal{F}[BWG_{nl}](k_x,k_y)|^2\mathrm{d}k_x\mathrm{d}k_y = 1$. Due to the orthogonality of the Hermite-Gauss eigenmodes one of the sums can be eliminated

$$\langle k_x^2\rangle = \sum_{m=0}^{\lfloor n/2\rfloor}\left[2^{-m}m!(n-2m)!\right]|p_m^{nl}|^2 \iint_{-\infty}^{\infty} k_x^2\left|\mathcal{F}[HG_{m,n-2m}](k_x,k_y)\right|^2 \mathrm{d}k_x\mathrm{d}k_y \tag{53}$$

The remaining integral can be solved using the relevant recursion relations of the Hermite polynomials $H_n(x)$

$$\iint_{-\infty}^{\infty} k_x^2\left|\mathcal{F}[HG_{m,n-2m}](k_x,k_y)\right|^2 \mathrm{d}k_x\mathrm{d}k_y = \frac{4}{w^2}(m+1/2). \tag{54}$$

Similarly, we find

$$\iint_{-\infty}^{\infty} k_y^2\left|\mathcal{F}[HG_{m,n-2m}](k_x,k_y)\right|^2 \mathrm{d}k_x\mathrm{d}k_y = \frac{2}{w^2}(n-2m+1/2), \tag{55}$$

and in total

$$\langle k_x^2\rangle + \langle k_y^2\rangle = \sum_{m=0}^{\lfloor n/2\rfloor}\left[2^{-m}m!(n-2m)!\right]|p_m^{nl}|^2\frac{(2n+3)}{w^2}. \tag{56}$$

This reduces even further due to normalization of the eigenvectors p_m^{nl}

$$\langle k_x^2\rangle + \langle k_y^2\rangle = \frac{2n+3}{w(z)^2}. \tag{57}$$

All that is left is to integrate over z to obtain the Gouy phase of the Boyer-Wolf Gaussian mode $BWG_{nl}(x,y)$

$$\phi_G^{0\to L} = -(n+3/2)\arctan(L/z_R), \tag{58}$$

which is the extra phase the beam acquires, due to it's momentum spread, during propagation from the beam waist to the distance L. The total Gouy-phase of one roundtrip in the Boyer-Wolf resonator is thus

$$\phi_G = -(2n+3)\arctan\left(\sqrt{L/(R_y-L)}\right). \tag{59}$$

Supplementary Note 5: Sextic Anharmonic Oscillator Polynomials

The sextic anharmonic oscillator refers to a potential energy function that is characterized by up to a sextic (sixth-degree) term in addition to the harmonic (quadratic) term. This sextic oscillator model is relevant in various areas in chemistry and physics. For example, the higher order sextic term is used to model the non-linearity of molecular potentials [11] or the anharmonic effects at high phonon energy levels [12]. In the previous section we have encountered such a sextic harmonic oscillator in the form of the eigenvalue equation

$$\left(-\partial_u^2 + k^2\omega^2 u^6 - Eu^2\right)\Phi(u) = \mu\Phi(u). \tag{60}$$

The eigenfunctions are obtained as

$$\Phi_{nl}(u) \propto u^n \exp(-\omega k u^4/4) P_{nl}\left(\left[2\sqrt{2\omega k}u^2\right]^{-1}\right), \tag{61}$$

where the parabolic polynomials are given by [3]

$$P_{nl}(u) = \sum_{m=0}^{\lfloor n/2 \rfloor} p_m^{nl} u^m. \tag{62}$$

The coefficients p_m^{nl} are obtained from the three-term recursion relation [3]

$$\frac{m+1}{\sqrt{2}} p_{m+1}^{nl} - \mu_l p_m^{nl} + \sqrt{2}(n+1-2m)(n+2-2m)p_{m-1}^{nl} = 0. \tag{63}$$

Note, the cofficients p_m^{nl} and the eigenvalue μ_l can be obtained from the diagonalization of a bidiagonal matrix which is given by the corresponding coefficients in Eq. (63). To be precise, p_m^{nl} are the components

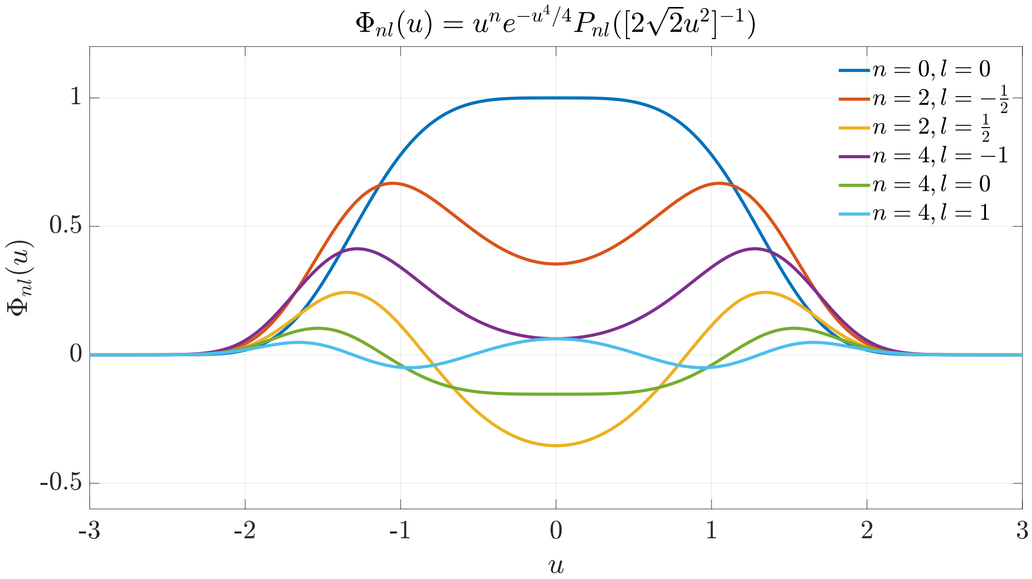

Supplementary Fig. 12 Even sextic harmonic oscillator eigenmodes. The functions $\Phi_{nl}(u)$, which are the product of the parabolic polynomials and a Gaussian, are used to construct the Boyer-Wolf Gaussian modes.

of the eigenvector with eigenvalue μ_l of the $(\lfloor n/2 \rfloor + 1) \otimes (\lfloor n/2 \rfloor + 1)$ matrix

$$M = \begin{pmatrix} 0 & \frac{1}{\sqrt{2}} & 0 & \cdots & 0 \\ \sqrt{2}(n-1)n & 0 & \frac{2}{\sqrt{2}} & & 0 \\ 0 & \sqrt{2}(n-3)(n-2) & 0 & \ddots & 0 \\ \vdots & & \ddots & \ddots & \frac{\lfloor \frac{n}{2} \rfloor}{\sqrt{2}} \\ 0 & 0 & 0 & \sqrt{2}(n+1-2\lfloor \frac{n}{2} \rfloor)(n+2-2\lfloor \frac{n}{2} \rfloor) & 0 \end{pmatrix} \tag{64}$$

In this construction care has to be exercised, so that $p_m^{nl} \in \mathbb{R}$, $p_0^{nl} > 0$ and the eigenvalues μ_l are sorted in ascending order, such that $\mu_{l_1} < \mu_{l_2} \Leftrightarrow l_1 < l_2$. As it was done in [3], we let the index label l run from $-\frac{1}{2}(\lfloor n/2 \rfloor)$ to $\frac{1}{2}(\lfloor n/2 \rfloor)$ in integer steps. Furthermore, we normalize the eigenvectors p_m^{nl}, so that

$$\sum_{m=0}^{\lfloor n/2 \rfloor} 2^{-m} m!(n-2m)! \left| p_m^{nl} \right|^2 = 1. \tag{65}$$

As an example, we show here the first parabolic polynomials up to $n = 6$

$$P_{0,0}(u) = P_{1,0}(u) = 1 \tag{66}$$

$$P_{2,\pm\frac{1}{2}}(u) = \pm u + \frac{1}{2} \tag{67}$$

$$P_{3,\pm\frac{1}{2}}(u) = \pm u + \frac{\sqrt{3}}{6} \tag{68}$$

$$P_{4,\pm 1}(u) = \frac{1}{2}u^2 \pm \frac{\sqrt{2}}{2}u + \frac{1}{8} \tag{69}$$

$$P_{4,0}(u) = -\frac{\sqrt{6}}{2}u^2 + \frac{\sqrt{6}}{24} \tag{70}$$

$$P_{5,\pm 1}(u) = \frac{\sqrt{6}}{4}u^2 \pm \frac{\sqrt{6}}{6}u + \frac{\sqrt{6}}{48} \tag{71}$$

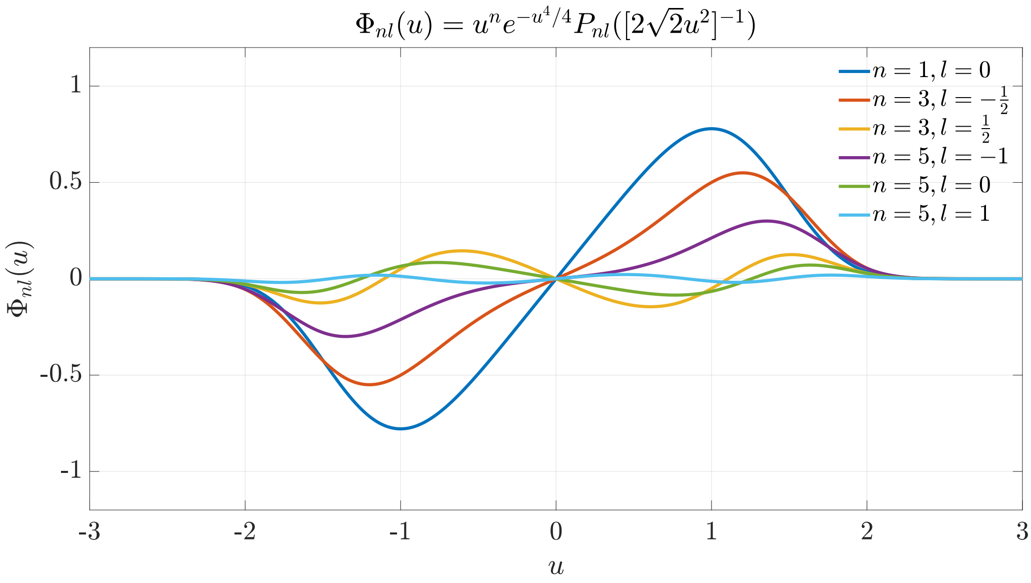

Supplementary Fig. 13 Odd sextic harmonic oscillator eigenmodes. The functions $\Phi_{nl}(u)$, which are the product of the parabolic polynomials and a Gaussian, are used to construct the Boyer-Wolf Gaussian modes.

$$P_{5,0}(u) = -\frac{\sqrt{5}}{2}u^2 + \frac{\sqrt{5}}{40} \tag{72}$$

$$P_{6,\pm\frac{3}{2}}(u) = \pm\sqrt{\frac{5-2\sqrt{5}}{15}}u^3 + \frac{1}{2}u^2 \pm \frac{1}{4}\sqrt{\frac{5+2\sqrt{5}}{15}}u + \frac{\sqrt{5}}{120} \tag{73}$$

$$P_{6,\pm\frac{1}{2}}(u) = \mp\sqrt{\frac{5+2\sqrt{5}}{15}}u^3 - \frac{1}{2}u^2 \pm \frac{1}{4}\sqrt{\frac{5-2\sqrt{5}}{15}}u + \frac{\sqrt{5}}{120}. \tag{74}$$

We plot some examples of the eigenfunctions of the sextic anharmonic oscillator, Eq. (60), in Supplementary Fig. (12,13).

Supplementary Note 6: Constant of Motion of the BWG Modes

The fundamental families of modes of the spherical resonator: HG-, LG- and IG-modes share the mathematically convenient property of being separable in their respective coordinate system and therefore feature a compact mathematical representation. Furthermore, the separability in each case is contingent on the existence of a special operator $\hat{S}$ that commutes with the 1:1 harmonic oscillator Hamiltonian $\hat{H}$. Therefore $\hat{H}$ and $\hat{S}$ share a common set of eigenvectors, which are the corresponding resonator modes. While the eigenvalues of $\hat{H}$ correspond to the Gouy phase shift, the eigenvalues of $\hat{S}$ correspond to an additional constant of motion. For example, the angular momentum operator $\hat{S}_J = x\partial_y - y\partial_x$ commutes with the 1:1 harmonic oscillator Hamiltonian, giving rise to the Laguerre-Gaussian modes. Naturally, the associated constant of motion is the orbital angular momentum j of LG modes, which is a physically relevant quantity and can be transferred to other physical systems [13].

In the previous section we have encountered the operator

$$\hat{S}_P = x\partial_x^2 - y\partial_x\partial_y - \frac{1}{2}\partial_x + k^2\omega^2 xy^2 \qquad \hat{S}_P BWG_{nl}(x,y) = \mu_{nl} BWG_{nl}(x,y) \tag{75}$$

which characterizes the symmetry of the 2:1 resonator. It can be shown by straight-forward calculation that $\hat{S}_P$ commutes with the 2:1 harmonic oscillator Hamiltonian $\hat{H}$ [3]. Therefore both operators share a common set of eigenstates or eigenmodes, which in this case are the BWG modes. Therefore the eigenvalues $\mu_{n,l}$ of the BWG with respect to $\hat{S}_P$ must correspond to a new constant of motion, which one may call the "parabolic" momentum. In Supplementary Fig. (14) we show a diagram of the parabolic momenta of the BWG modes.

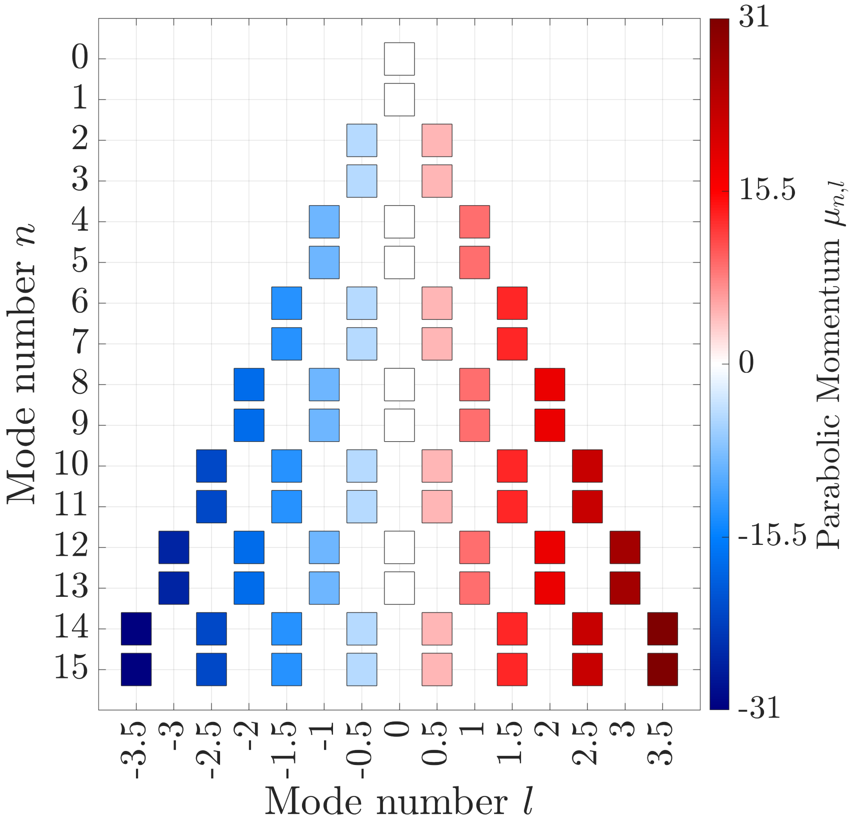

Supplementary Fig. 14 Diagram of the parabolic momentum of the BWG modes. We show the eigenvalues, or parabolic momenta, $\mu_{n,l}$ of the BWG modes with the parabolic symmetry operator $\hat{S}_P$. Here we use normalized units as in [3].

Supplementary Note 7: 2:1 Anisotropic GRIN Medium

Another possibility to observe the Boyer-Wolf-Gaussian beams is inside a 2:1 anisotropic gradient-refractive-index (GRIN) medium. We start by considering the paraxial wave equation, which describes the evolution of a beam of light in a dielectric medium

$$2kn_0 i\partial_z\psi = -\nabla_\perp^2\psi - k^2\left(n^2 - n_0^2\right)\psi, \tag{76}$$

where $\psi = \psi(x, y, z)$ is the electric field envelope, $\nabla_\perp = (\partial_x, \partial_y)^T$ and (x, y) are the transverse coordinates to the propagation direction z. Further, $k = 2\pi/\lambda$ is the vacuum wavenumber, $n = n(x, y, z)$ the refractive index profile and n_0 is the reference refractive index of the medium. We now study the specific case of an astigmatic GRIN medium that exhibits the refractive index profile

$$n^2(x, y) = n_0^2\left[1 - a^2\left((2x)^2 + y^2\right)\right], \tag{77}$$

which we depict in Supplementary Fig. (15). Evidently, this is a 2D harmonic oscillator potential, with twice the strength (or confinement) in x-direction. In short, we obtain the full equation of motion

$$2kn_0 i\partial_z\psi = -(\partial_x^2 + \partial_y^2)\psi + k^2 n_0^2 a^2\left((2x)^2 + y^2\right)\psi. \tag{78}$$

We separate the z-dependence, i.e. $\psi(x, y, z) = \exp(-iEz/(2kn_0))\phi(x, y)$, yielding the eigenvalue equation

$$-(\partial_x^2 + \partial_y^2)\phi + k^2 n_0^2 a^2\left(4x^2 + y^2\right)\phi = E\phi. \tag{79}$$

which is identical to Eq. (22), after defining $\omega \coloneqq n_0 a$. Therefore, the Boyer-Wolf-Gaussian modes are also eigenmodes of the 2:1 anisotropic GRIN medium

$$BWG_{nl}(u, v, z) = c_{nl}\left(\sqrt{2}\frac{uv}{w_\sigma}\right)^n \exp\left(-\frac{u^4 + v^4}{2w_\sigma^2}\right) P_{nl}\left(\left[4\frac{u^2}{w_\sigma}\right]^{-1}\right) P_{nl}\left(-\left[4\frac{v^2}{w_\sigma}\right]^{-1}\right) e^{-i\beta z} \tag{80}$$

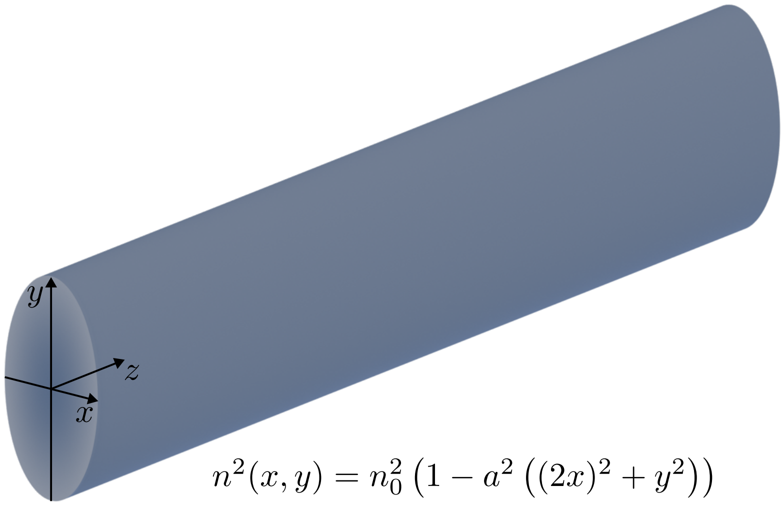

Supplementary Fig. 15 Anisotropic 2:1 GRIN medium implementing the 2:1 2D anisotropic oscillator potential.

$$\beta = a(n + 3/2), \tag{81}$$

where the width of the modes is $w_\sigma^2 = 2/(kn_0 a)$. This is indeed what we observe in Supplementary Fig. (16), where we show 5 examples that clearly remain invariant during propagation. For comparison we simulate the propagation of the same 5 modes in free space in Supplementary Fig. (17,18), where we observe diffraction as expected.

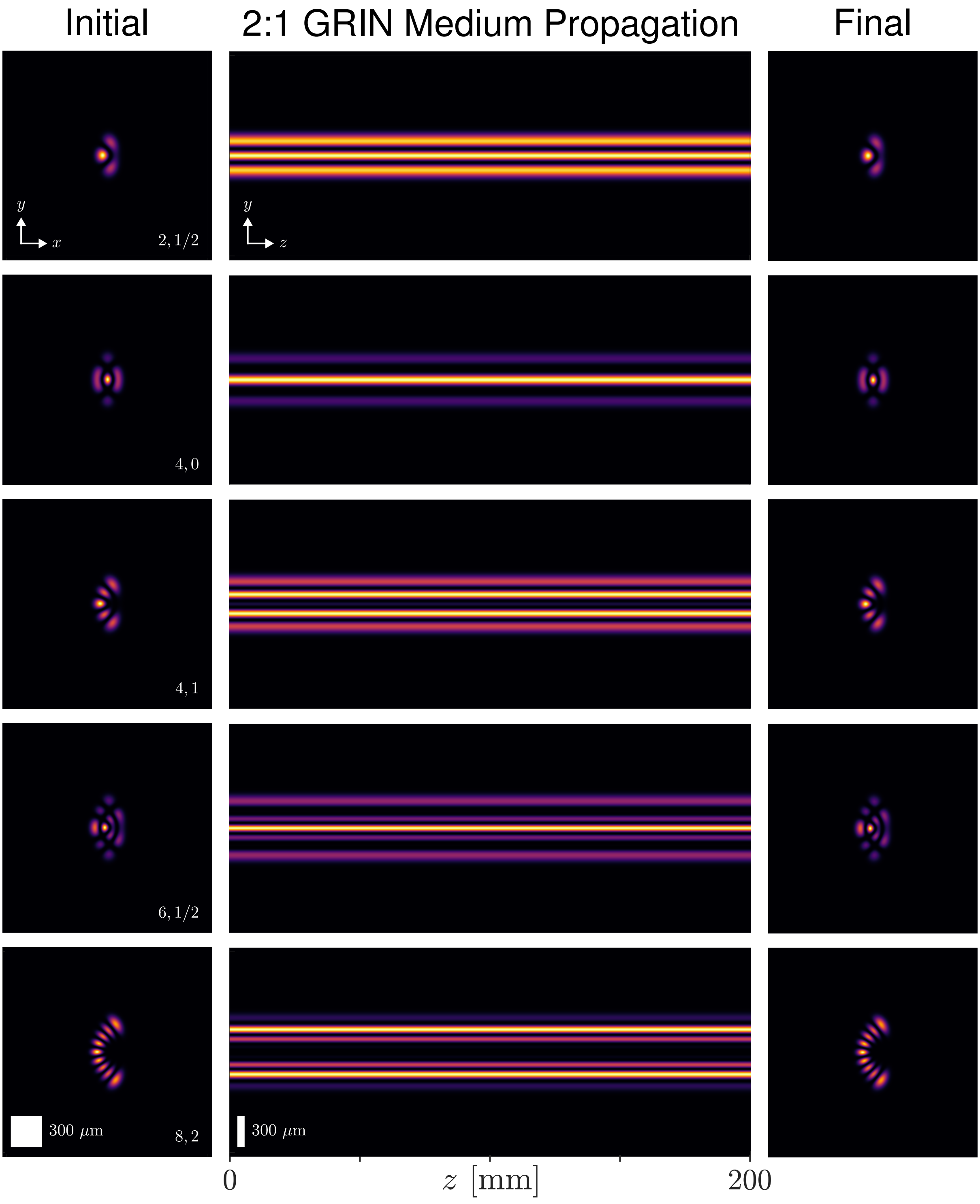

Supplementary Fig. 16 Simulation of the propagation of several Boyer-Wolf-Gaussian modes in the 2:1 anisotropic GRIN medium. The left-most panels show the initial intensities, the center panels the $y-z$ slice at $x=0$. In the simulations we choose the wavelength $\lambda = 1064$ nm and the GRIN-curvature parameter $a = 0.0546$ cm^{-1} and reference refractive index $n_0 = 1$.

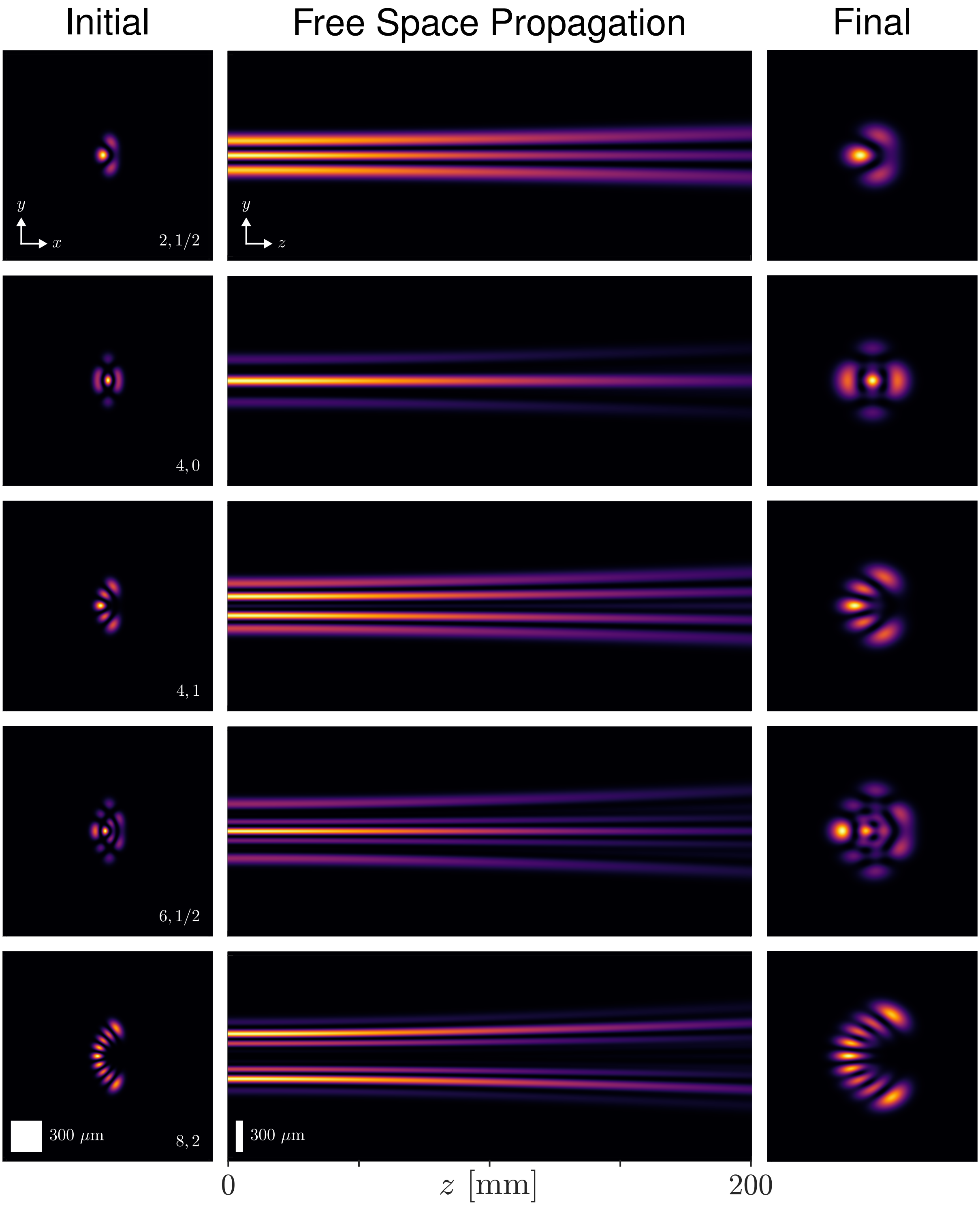

Supplementary Fig. 17 Simulation of the propagation of several Boyer-Wolf-Gaussian modes in free space. The left-most panels show the initial intensities, the center panels the $y-z$ slice at $x=0$ of the propagation and the right-most panel shows the final intensities. In the simulations we choose the wavelength $\lambda = 1064$ nm.

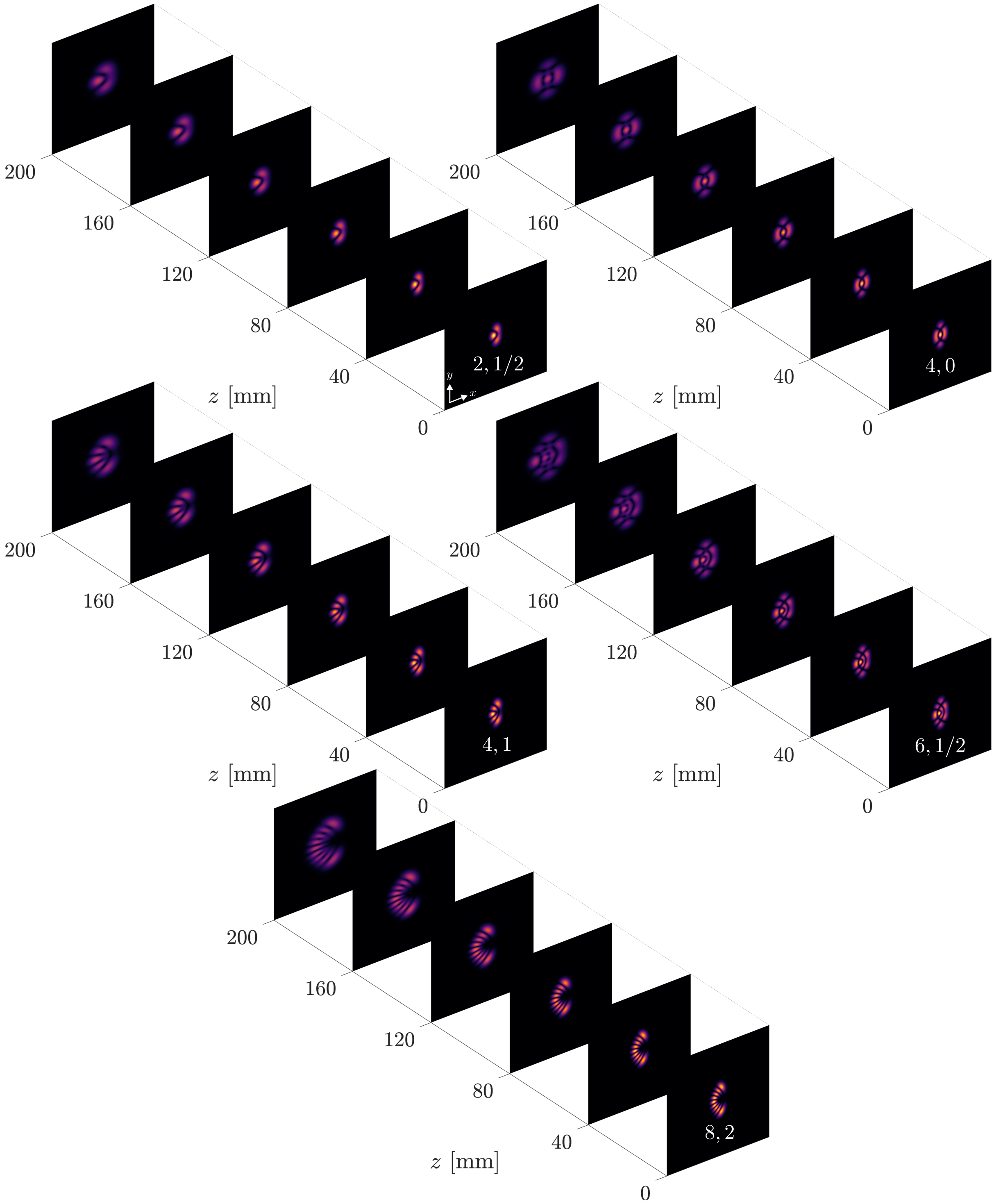

Supplementary Fig. 18 Simulation of the propagation of several Boyer-Wolf-Gaussian modes in free space. Here we show the same simulations as in Supplementary Fig. (17) but as several x-y-slices along propagation.

Supplementary Note 7.1: Self-Healing Effect of BWG Modes in a 2:1 GRIN Medium

Self-healing is an intriguing property of non-diffractive beams [14, 15], whereby such beams restore their amplitude profile during propagation after encountering an obstacle. In a recent work, J. Jia et. al. [16] have demonstrated theoretically and experimentally the self-healing of structured beams in lens-like media. They show that the self-healing effect is a result of a superposition of fundamental traveling waves and the characteristics of ray propagation in inhomogeneous lens-like media with a quadratic gradient index. Specifically, they study the shadows, created by obstacles, which travel along sinusoidal paths inside a lens-like GRIN medium following geometrical ray theory. As we have shown in the main manuscript, the BWG modes can be transformed into non-diffractive Weber beams [17] by increasing the mode number n and mode width w to infinity, while keeping the ratio $k_t = 2\sqrt{n/w}$ constant. Furthermore, the BWG modes are also eigenmodes of the 2:1 GRIN medium. Thus it is expected that also BWG modes exhibit self-healing abilities as demonstrated in ref. [16]. To investigate this behavior we follow Ref. [16] and study the propagation of the $n = 44, l = 0$ BWG mode in a 2:1 GRIN medium with $a = 1.1272$ 1/m. We block out from the initial field a small circle of radius $r = 0.3$ mm at a distance of $x = 1$ mm away from the origin along the x-axis. In Supplementary Fig. (19) we show the $x - z$ slice of the propagation until a distance of $L_z = 2.786$ m. We observe the emergence of shadows that follow a sinusoidal propagation path and reform the initial blocked circle at $z = L_z$. In Supplementary Fig. (20) we show the $x - y$-slices at several steps of the propagation. At $z = L_z/2$ we observe only a partial recurrence of the circle at position $-x$, which is explained by the fact that the $x-$ and $y-$ focal planes of the 2:1 GRIN medium are not at the same distance from the origin $z = 0$. However, at $z = L_z/4$ and $z = 3L_z/4$ we observe the maximum amount of healing of the BWG mode from the circular obstacle, in accordance with the findings in Ref. [16], thus showing that BWG modes exhibit the very appealing self-healing property.

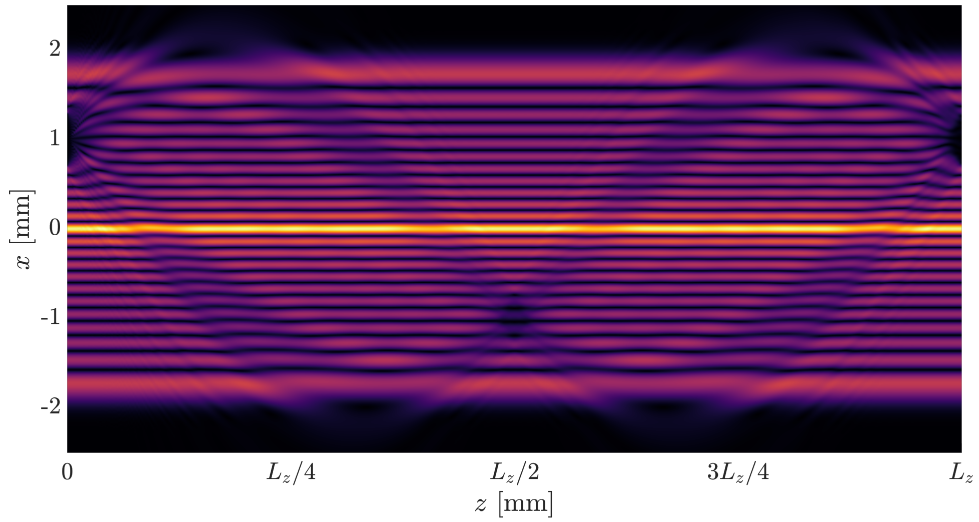

Supplementary Fig. 19 Propagation of partially blocked high-order BWG mode We show the $x - z$-slice of the propagation of the $n = 44, l = 0$ BWG mode in a 2:1 GRIN medium with $a = 1.1272$ 1/m and $\lambda = 1064$ nm. The blocked region causes the emergence of shadows that propagate along sinusoidal paths and reconstitute the initial blocked circle at $z = L_z = 2.786$ m.

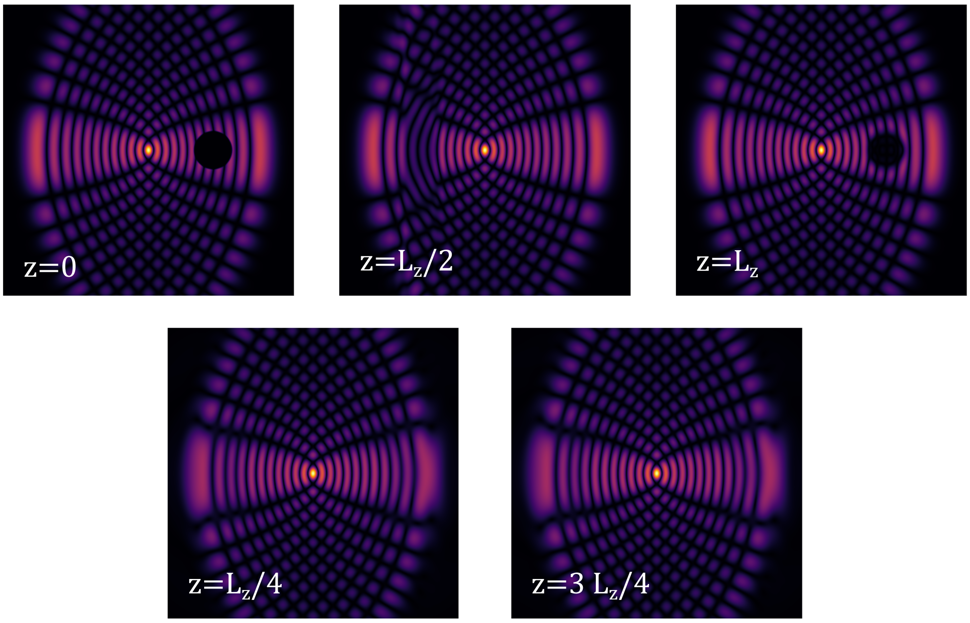

Supplementary Fig. 20 Partial and fully self-healing of higher order BWG modes We show several $x-y$-slices of the propagation shown in Supplementary Fig. (19). At $z = L_z/4$ and $z = 3L_z/4$ we observe (almost) complete healing of the beam profile after encountering the circular block. At other distances we observe only partial healing or, in the extreme case, the recurrence of the initial circular block.

Supplementary Note 8: Lasing threshold of the Boyer-Wolf 2:1 anisotropic laser resonator

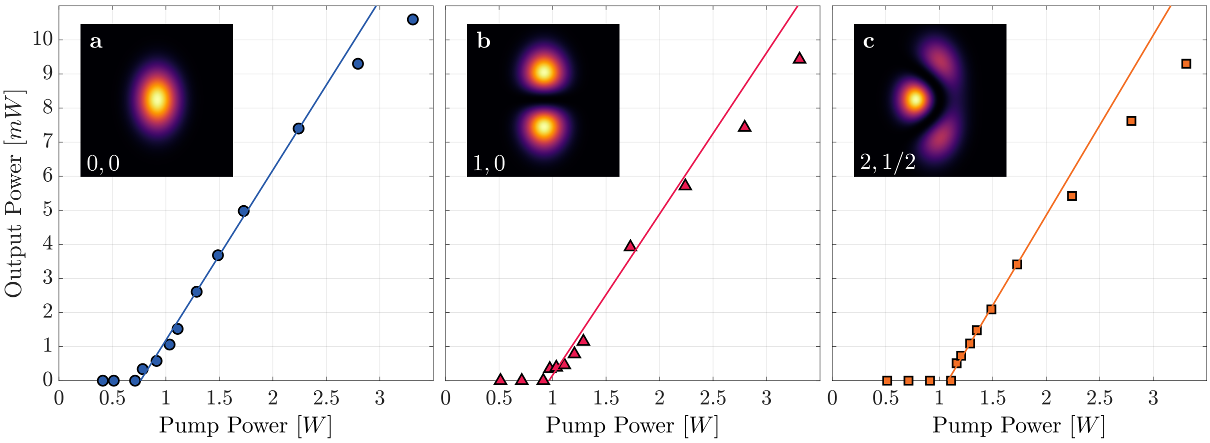

Supplementary Fig. 21 Lasing threshold of the first three BWG modes in our BWG resonator. We plot the measured output power as a function of the input pump power when the cavity is configured to lase in the modes **a** $n, l = 0, 0$, **b** $n, l = 1, 0$ and **c** $n, l = 2, 1/2$. We observe that the threshold increases with the mode order n. For pump powers between 2 to 3 W, we observe the onset of saturation.

Supplementary Note 9: Cavity Alignment and Configuration to Excite Higher Order BWG Modes

In order to excite higher order BWG modes we have tried cavity lengths L between 11 cm and 30 cm. In Supplementary Tab. (1) we show the cavity length which yielded the best lasing mode. In general, it is possible to excite higher order modes by increasing the pump spot size and keeping the cavity length fixed. However, in our case it was more convenient to leave the pump spot size invariant and to change the cavity length, which is directly related to the mode size in Eq. (43), instead. Note that care has to be exercised, so that the lens is always at the center of the cavity, whenever changing its length. As a result, higher order modes were usually achieved with shorter cavity lengths. For each cavity length we adjusted the horizontal and vertical tilt-angles of the cylindrical mirror and lens, which amounts to 4 degrees of freedom. Each tilt-angle determines the orientation of the optical axis of the mirror and lens with respect to the flat output-coupling mirror. In general, a larger tilt angle was associated with a higher number of lobes and width in the corresponding direction. For example, when starting from the fundamental $(0, 0)$ mode, it was possible to increase the horizontal (vertical-) tilt of the cylindrical mirror to add additional lobes and to "stretch" the mode in the horizontal (vertical-) direction. We have opted to use the mirror for the coarse-grained tilt-adjustments and only adjust the lens-tilt for fine-tuning to achieve the desired modes.

Mode (n,l)	L [cm]	w_0 [μm]
(0,0)	20	260
(1,0)	20	260
(2,1/2)	20	260
(3,1/2)	20	260
(4,0)	17	259
(4,1)	20	260
(5,0)	17	259
(5,1)	20	260
(6,1/2)	17	259
(6,3/2)	20	260
(7,1/2)	15.5	257
(7,3/2)	17	259
(8,0)	17	259
(8,1)	11	246
(8,2)	20	260
(9,0)	15.5	257
(9,1)	11	246
(9,2)	20	260

Supplementary Table 1 Cavity lengths L used to obtain the corresponding (n, l) BWG modes depicted in Supplementary Fig. (3) of the main manuscript. We also show the theoretically expected mode waist size w_0, according to Eq. (43).

Supplementary Note 10: Mode Selection via Cavity Misalignment

In the following we provide a brief theoretical analysis to corroborate the mode selection mechanism due to the cavity misalignment. We begin by noting that a misaligned ABCD element can be modeled by a 3×3 ABCDEF matrix [18]

$$M = \begin{pmatrix} A & B & \Delta x \\ C & D & \Delta s_x \\ 0 & 0 & 1 \end{pmatrix}, \tag{82}$$

where Δx is the displacement of the element in the transverse direction and Δs_x is the tilt angle with respect to the optical axis. The round-trip matrix in a Fabry-Pérot cavity with a displaced and misaligned spherical mirror is thus

$$\hat{M} = \begin{pmatrix} 1 & L & 0 \\ 0 & 1 & 0 \\ 0 & 0 & 1 \end{pmatrix} \begin{pmatrix} 1 & 0 & \Delta x \\ -2/R & 1 & \Delta s_x \\ 0 & 0 & 1 \end{pmatrix} \begin{pmatrix} 1 & L & 0 \\ 0 & 1 & 0 \\ 0 & 0 & 1 \end{pmatrix} = \begin{pmatrix} 2g-1 & 2gR(1-g) & \Delta x + L\Delta s_x \\ -2/R & 2g-1 & \Delta s_x \\ 0 & 0 & 1 \end{pmatrix}. \tag{83}$$

M can be now decomposed into the following product

$$M = \underbrace{\begin{pmatrix} 1 & 0 & a \\ 0 & 1 & b \\ 0 & 0 & 1 \end{pmatrix}}_{=\hat{T}} \underbrace{\begin{pmatrix} \cos\alpha & 1/\omega \sin\alpha & 0 \\ -\omega \sin\alpha & \cos\alpha & 0 \\ 0 & 0 & 1 \end{pmatrix}}_{=\hat{R}}, \tag{84}$$

where $\hat{R}$ is a rotation (including a scaling of the ray angle by ω), $\hat{T}$ is a translation, $\alpha = 4L\omega$, $\omega = [L(R-L)]^{-1/2}$, $a = \Delta x + L\Delta s_x$ and $b = \Delta s_x$. It is straight-forward to find the matrix logarithms of $\hat{R}$ and $\hat{T}$

$$\hat{W} = \log \hat{T} = \begin{pmatrix} 0 & 0 & a \\ 0 & 0 & b \\ 0 & 0 & 0 \end{pmatrix} \qquad \hat{Q} = \log \hat{R} = \begin{pmatrix} 0 & \alpha/\omega & 0 \\ -\alpha\omega & 0 & 0 \\ 0 & 0 & 0 \end{pmatrix}. \tag{85}$$

Thus, we have the first result

$$\hat{M} = \exp\left(\hat{W}\right) \exp\left(\hat{Q}\right). \tag{86}$$

We now use the BCH formula in order to combine the two operator exponentials

$$\begin{aligned} \hat{M} = \exp\Big(\hat{W} + \hat{Q} + \frac{1}{2}\underbrace{\left[\hat{W}, \hat{Q}\right]}_{=-\hat{V}} + \frac{1}{12}\underbrace{\left[\hat{W}, \left[\hat{W}, \hat{Q}\right]\right]}_{=0} + \frac{1}{12}\underbrace{\left[\hat{Q}, \left[\hat{Q}, \hat{W}\right]\right]}_{-\alpha^2 W} \\ - \frac{1}{24}\underbrace{\left[\hat{Q}, \left[\hat{W}, \left[\hat{W}, \hat{Q}\right]\right]\right]}_{=0} + \dots\Big), \end{aligned} \tag{87}$$

where

$$\hat{V} = \begin{pmatrix} 0 & 0 & \frac{\alpha b}{\omega} \\ 0 & 0 & -\alpha a \omega \\ 0 & 0 & 0 \end{pmatrix}. \tag{88}$$

We neglect higher order commutators and find

$$\hat{M} \approx \exp\left(\hat{Q} - \frac{1}{2}\hat{V} + \left(1 - \frac{1}{12}\alpha^2\right)\hat{W}\right). \tag{89}$$

After one period $z = 2L$ we thus must have

$$\hat{M} = \exp\left(2L\left(\tilde{Q} - \frac{1}{2}\tilde{V} + \left(1 - \frac{1}{12}\alpha^2\right)\tilde{W}\right)\right), \tag{90}$$

where we defined the scaled operators $\tilde{Q} = \hat{Q}/(2L)$, $\tilde{V} = \hat{V}/(2L)$ and $\tilde{W} = \hat{W}/(2L)$. Now we can write down the continuous equations of motion that emulate the action of $\hat{M}$

$$\begin{pmatrix} \dot{x} \\ \dot{s}_x \\ 0 \end{pmatrix} = \left[\tilde{Q} - \frac{1}{2}\tilde{V} + \left(1 - \frac{1}{12}\alpha^2\right)\tilde{W}\right]\begin{pmatrix} x \\ s_x \\ 1 \end{pmatrix} = \begin{pmatrix} 0 & 2 & -2s_0 \\ -2\omega^2 & 0 & 2\omega^2 x_0 \\ 0 & 0 & 0 \end{pmatrix}\begin{pmatrix} x \\ s_x \\ 1 \end{pmatrix}, \tag{91}$$

with $s_0 = (b + \frac{a}{2L}(\frac{4}{3}L^2\omega^2 - 1))/2$ and $x_0 = (a - \frac{b}{2L\omega^2}(\frac{4}{3}L^2\omega^2 - 1))/2$. In short,

$$\dot{x} = 2s_x - 2s_0 \tag{92}$$

$$\dot{s}_x = -2\omega^2 x + 2\omega^2 x_0. \tag{93}$$

Using Hamilton's equations

$$\dot{x} = \frac{\partial H}{\partial s_x} \tag{94}$$

$$\dot{s}_x = -\frac{\partial H}{\partial x} \tag{95}$$

we find the effective Hamiltonian

$$H = s_x^2 - 2s_0 s_x + \omega^2 x^2 - 2\omega^2 x_0 x. \tag{96}$$

We can rewrite this as

$$H = (s_x - s_0)^2 - s_0^2 + \omega^2 (x - x_0)^2 - \omega^2 x_0^2. \tag{97}$$

Now we substitute $s_x \to -i\partial_x/k$

$$\hat{H} = \left(\frac{i}{k}\partial_x + s_0\right)^2 - s_0^2 + \omega^2 (x - x_0)^2 - \omega^2 x_0^2, \tag{98}$$

and multiply both sides with k^2 to obtain

$$\hat{H} = (i\partial_x + ks_0)^2 - k^2 s_0^2 + k^2\omega^2 (x - x_0)^2 - k^2\omega^2 x_0^2, \tag{99}$$

where we found the constant gauge field $A = ks_0$, which we gauge away with $\psi = \phi e^{-i\Lambda}$ and the choice $-\partial_x \Lambda = ks_0 \Leftrightarrow \Lambda = -ks_0 x$. The potential V remains unmodified, since $-\partial_z \Lambda = 0$. We have arrived at

$$\hat{H} = -\partial_x^2 + k^2\omega^2 (x - x_0)^2 - k^2\omega^2 x_0^2 - k^2 s_0^2. \tag{100}$$

In summary, the misaligned mirror displaces the associated harmonic oscillator potential by the amount x_0, modifies the Gouy phase shift by $-k^2\omega^2 x_0^2 - k^2 s_0^2$ and the gauge-factor e^{iks_0x} indicates a linear tilt of the phase-fronts at the flat output mirror. It is straight-forward to extend this analysis to our anisotropic 2:1 cavity, where the misaligned mirror and lens produce a shift in the transverse position (x, y) and Gouy phase and a tilt of the phase front of the BWG modes. To illustrate the role of the misalignment in the mode selection mechanism we now perform the following simulations. We assume a Gaussian pump-spot (radius 250 μm) and displace it by (x, y), which is equivalent to displacing the modes, and calculate the overlap between the pump and all BWG and HG modes up to $n = 9$ ($R = 40$ cm, $L = 20$ cm, $\lambda = 1064$ nm). Then we find, for each displacement (x, y), the BWG mode and HG mode with the highest overlap. We plot the difference of the overlaps between the best BWG and HG mode in Supplementary Fig. 22, so that red (blue) regions indicate a higher overlap for BWG (HG) modes. As one can see, there are regions where the BWG modes dominate over HG modes and vice versa. The central white region corresponds to the fundamental $n = 0$ mode, which is the same for BWG and HG modes. Our strategy to excite BWG modes thus amounts to exploring this space by changing the tilt angles of the mirror and lens until a red region is found. The real shape and position of these regions strongly depend on the cavity parameters R, L, the pump spot size and also any imperfections of the experimental setup. Thus a certain degree of trial and error is required in order to excite BWG modes in our setup.

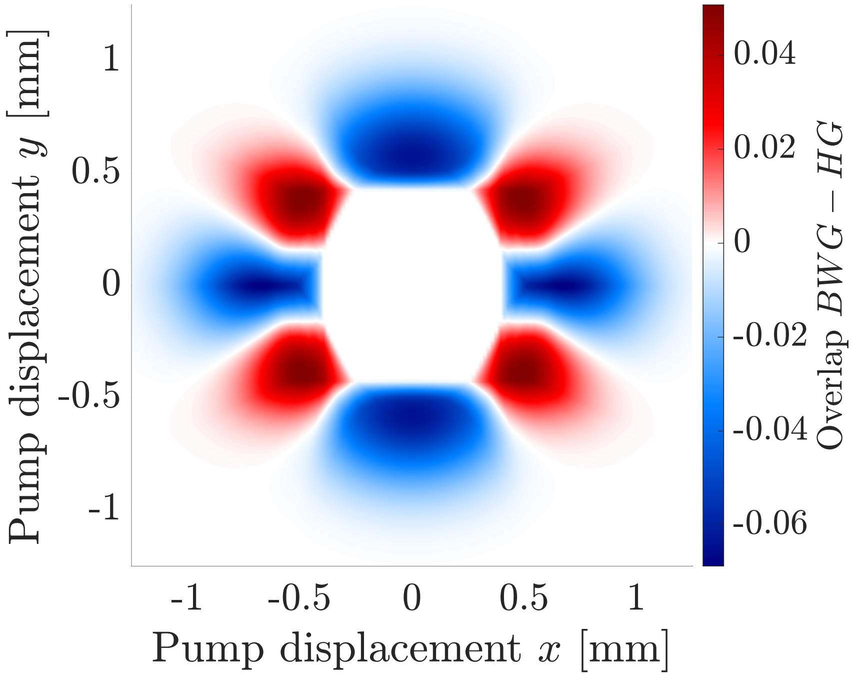

Supplementary Fig. 22 Difference of the overlaps between the pump spot and the BWG and anisotropic HG modes We displaced the Gaussian pump-spot (radius 250 μm) by (x, y) and calculated the overlap between the pump and all BWG and HG modes up to $n = 9$. Then we find, for each displacement (x, y), the BWG- and HG-mode with the highest overlap. We plot the difference of the overlaps between the best BWG and HG mode, so that red (blue) regions indicate a higher overlap for BWG (HG) modes.

References

[1] Kogelnik, H., Li, T.: Laser beams and resonators. Appl. Opt. **5**(10), 1550–1567 (1966)

[2] Sommer, A., Simon, J.: Engineering photonic floquet hamiltonians through fabry–pérot resonators. New Journal of Physics **18**(3), 035008 (2016)

[3] Boyer, C.P., Wolf, K.B.: The 2:1 anisotropic oscillator, separation of variables and symmetry group in Bargmann space. Journal of Mathematical Physics **16**(11), 2215–2223 (2008)

[4] Friš, J., Mandrosov, V., Smorodinsky, Y.A., Uhlíř, M., Winternitz, P.: On higher symmetries in quantum mechanics. Physics Letters **16**(3), 354–356 (1965)

[5] Lévai, G., Ishkhanyan, A.M.: Exact solutions of the sextic oscillator from the bi-confluent heun equation. Modern Physics Letters A **34**(18), 1950134 (2019)

[6] Dutta, A., Willey, R.: Exact analytic solutions for the quantum mechanical sextic anharmonic oscillator. Journal of mathematical physics **29**(4), 892–900 (1988)

[7] Sous, A.: Solution for the eigenenergies of sextic anharmonic oscillator potential v (x)= a6x6+ a4x4+ a2x2. Modern Physics Letters A **21**(21), 1675–1682 (2006)

[8] Znojil, M.: Sextic-oscillator puzzle and its solution. Physical Review D **34**(4), 1224 (1986)

[9] Truong, T.: Weyl quantization of anharmonic oscillators. Journal of Mathematical Physics **16**(5), 1034–1043 (1975)

[10] Feng, S., Winful, H.G.: Physical origin of the gouy phase shift. Opt. Lett. **26**(8), 485–487 (2001)

[11] Császár, A.G.: Anharmonic molecular force fields. WIREs Computational Molecular Science **2**(2), 273–289 (2012)

[12] Qiu, Y., Wu, C.Q., Nasu, K.: Dual electron-phonon coupling model for gigantic photoenhancement of the dielectric constant and electronic conductivity in $SrTio_3$. Phys. Rev. B **72**, 224105 (2005)

[13] Allen, L., Beijersbergen, M.W., Spreeuw, R.J.C., Woerdman, J.P.: Orbital angular momentum of light and the transformation of laguerre-gaussian laser modes. Phys. Rev. A **45**, 8185–8189 (1992)

[14] Gutiérrez-Vega, J.C., Iturbe-Castillo, M.D., Ramírez, G.A., Tepichín, E., Rodríguez-Dagnino, R.M., Chávez-Cerda, S., New, G.H.C.: Experimental demonstration of optical mathieu beams. Optics Communications **195**(1), 35–40 (2001)

[15] Bandres, M.A., Gutiérrez-Vega, J.C., Chávez-Cerda, S.: Parabolic nondiffracting optical wave fields. Opt. Lett. **29**(1), 44–46 (2004)

[16] Jia, J., Lin, H., Fu, S., Gómez-Correa, J.E., Li, Z., Chen, Z., Chávez-Cerda, S.: Shadows of structured beams in lenslike media. Opt. Express **31**(24), 40824–40835 (2023)

[17] Zhang, P., Hu, Y., Li, T., Cannan, D., Yin, X., Morandotti, R., Chen, Z., Zhang, X.: Nonparaxial mathieu and weber accelerating beams. Phys. Rev. Lett. **109**, 193901 (2012)

[18] Siegman, A.E.: Lasers, pp. 607–614. University Science Books
